# Modulating pressure in the Orbitrap improves sensitivity and mass resolution in charge detection mass spectrometry

**DOI:** 10.1038/s41467-026-76691-1

**Published:** 2026-08-13

**Authors:** Eduard H.T.M. Ebberink, Elena Giaretta, Arjan Barendregt, Jan Fiala, Victor C. Yin, Tobias P. Wörner, Kyle L. Fort, Alexander A. Makarov, Albert. J. R. Heck

**Affiliations:** 1 Biomolecular Mass Spectrometry and Proteomics, Bijvoet Center for Biomolecular Research and Utrecht Institute for Pharmaceutical Sciences, Utrecht University, Utrecht, The Netherlands; 2 Netherlands Proteomics Center, Utrecht, The Netherlands; 3 Thermo Fisher Scientific (Bremen) GmbH, Bremen, Germany; 4 Present Address: Department of Pharmacy, University of Copenhagen, Copenhagen, Denmark

**Keywords:** Mass spectrometry, Bioanalytical chemistry, Biochemistry

## Abstract

Native mass spectrometry has become a key method for studying macromolecular assemblies, providing insights into structures, stoichiometries, and binding interactions. A key aspect for the transmission of electrospray-generated bioparticles into the mass analyzer is the use of gas for collisional cooling and ion desolvation. However, in Orbitrap-based mass spectrometry, the elevated pressure may negatively affect ions, as collisions with background gas can destabilize ion trajectories, potentially leading to incorrect mass determination. These effects are amplified when the pressure in the collision cell is high, as required for large biological assemblies, and when ions are measured for (ultra)long acquisition times in the Orbitrap, as required for high-resolution mass spectrometry. To address these issues, we modified a standard Q Exactive™ UHMR by installing a pulsed valve to control gas flow and limit gas leakage into the Orbitrap. This way, ion transmission and desolvation are maintained while the ultrahigh vacuum in the mass analyzer is enhanced during acquisition. We show that this improves ion detection of various assemblies, including adeno-associated viruses, IgM, and plasmid DNA, with superior mass accuracy and resolving power. The pulsed valve implementation will benefit nearly all mass measurements, setting the stage for next-generation Orbitrap-based, single-ion mass spectrometry.

## Abstract (web summary)

Orbitrap-based single-ion charge mass spectrometry of biomolecular assemblies can be hampered by gas-induced ion losses. Here, the authors introduce a pulsed valve to regulate vacuum conditions resulting in improved ion detection enabling sensitive, accurate, and higher-resolution mass measurements

## Introduction

Native mass spectrometry (MS) has become an essential tool for interrogating the architecture of proteins, protein assemblies, and other biomolecular complexes^1–5^. Allowing biomolecules to retain their physiological, native state (regardless of their composition of either proteins, peptides, nucleotides, and/or lipids) and accurately determining their mass has proven to be beneficial in the study of ligand binding, structural conformations, and stoichiometries of protein assemblies as well as proteoform profiling of complex glycoproteins and biopharmaceuticals^6^. As with most MS applications, the introduction of external gas to the mass spectrometers’ vacuum system is a vital part of native MS, especially for the detection of large biomolecular complexes. Following electrospray ionization (ESI) of analytes and conversion from an aqueous solution to the gas phase, the ionized particles retain solvent and exhibit velocity spread. Therefore, the use of inert gases at specific sites along the flight path is essential for desolvation, removal of small adducts, and focusing and collisional cooling of ions along their trajectory to the mass analyzer to improve transmission^7–10^. While gas can be delivered close to the ion source, Orbitrap^TM^-based mass spectrometers achieve most of the ion desolvation and collisional cooling by providing gas into the higher-energy collision-induced dissociation (HCD) cell^11,12^. Together with additional electronic modifications to improve the transmission of high-mass ions (e.g., lowering quadrupole radio frequencies), Orbitrap-based platforms such as the Q Exactive^TM^ UHMR instrument have become the go-to mass spectrometers in this thriving research field^13,14^. The implementation of external gas therein is a key factor in enabling MS measurement of a wide range of native macromolecular bioparticles and pharmaceuticals^14–19^.

Because optimized instrumentation for native MS allows for detection of bigger and heterogeneous bioparticles, acquired *m/z*-spectra often exhibit intrinsic mass heterogeneity (protein isoforms, variable stoichiometries, extensive glycosylation, and post-translational modifications), which leads to dense spectral features from which the charge states cannot be resolved anymore from consecutive *m/z* peaks^20^. In recent years, single-molecule charge-detection mass spectrometry (CDMS) has proved useful to alleviate this issue. By detecting single ions and determining charge from transient recordings’ amplitudes, the mass of each individual ion can be established, sidelining heterogeneity issues in bulk ion deconvolution^21–23^. CDMS is particularly effective in enabling native MS on heterogeneous, high-molecular-weight samples such as recombinant adeno-associated viruses (rAAVs), DNA plasmids, and lipid nanoparticles^24–27^. And with the availability of commercial CDMS software for Orbitrap mass spectrometers (Direct Mass Technology) and liquid chromatography timescale coupling^28–32^, a more straightforward implementation of CDMS into pharmaceutically relevant workflows, both in academia and industry, is now attainable^33–36^.

With accurate charge determination (inferred from the ions’ signal amplitude in the raw transient recording) being essential for CDMS^37^, we recently introduced modifications that enabled us to record ultralong transients with acquisition times up to ~25 s (compared to conventional milliseconds-to-seconds-long recording times)^38^. We demonstrated that these ultralong transients dramatically decreased charge uncertainty and, in addition, improved signal-to-noise ratio and mass resolution, culminating in superior separation of mass species. Importantly, optimal CDMS detection requires the signal from individual ions to remain stable throughout the entire recording time^39,40^. Particle instability is mainly caused by collisions with background gas in the Orbitrap mass analyzer that originates from the externally supplied collision gas. Such background gas collisions result in dramatic shifts or gradual drifting in oscillation frequency and occasionally lead to the complete loss of the ion^40^. Signals that display such artifacts are typically filtered out and removed from subsequent mass analysis; however, ion stability is not equal for all samples, which can introduce bias and lead to spurious results in CDMS quantification and mass determination^41^. Especially for smaller biomolecular particles that oscillate at a high frequency in the Orbitrap mass analyzer, ion trajectories may be especially unstable^38,40^. For such low *m/z*, high-frequency ions, the traveling distance and the number of accompanying collisions with residual gas molecules are high, and due to their low mass, the impact of such collisions is elevated^40,42^. Therefore, while external gas supplied to the collision cell is essential for ion desolvation and collisional cooling, it can also be detrimental when acquiring long transient recordings.

So far, the optimal settings for Orbitrap native MS and CDMS are inherently in conflict: elevated gas pressure in the HCD cell is needed for optimal ion focusing and transmission, but this raises the pressure in the ultrahigh vacuum (UHV) of the Orbitrap analyzer, negatively affecting ion stability during mass analysis. Here, we address and resolve this mismatch by implementing an active temporal-pressure-modulation strategy using a pulsed-valve system placed between the gas source and the HCD cell. This enables us to secure the beneficial aspects of each pressure regime (high and low) for sufficient ion transmission and optimal stability in the Orbitrap mass analyzer. We leverage this improved performance to measure a broad range of biopharmaceutical samples, demonstrating key advantages in ion stability, resolution, and mass and charge accuracy with the pulsed valve. Therefore, we suggest that this gas control system would be beneficial not only for Orbitrap-based CDMS, but potentially in the much broader context of all protein- and peptide-centric mass analyses.

## Results

### Implementation of a pulsed valve to restrict gas leakage into the Orbitrap analyzer

**Fig. 1:**
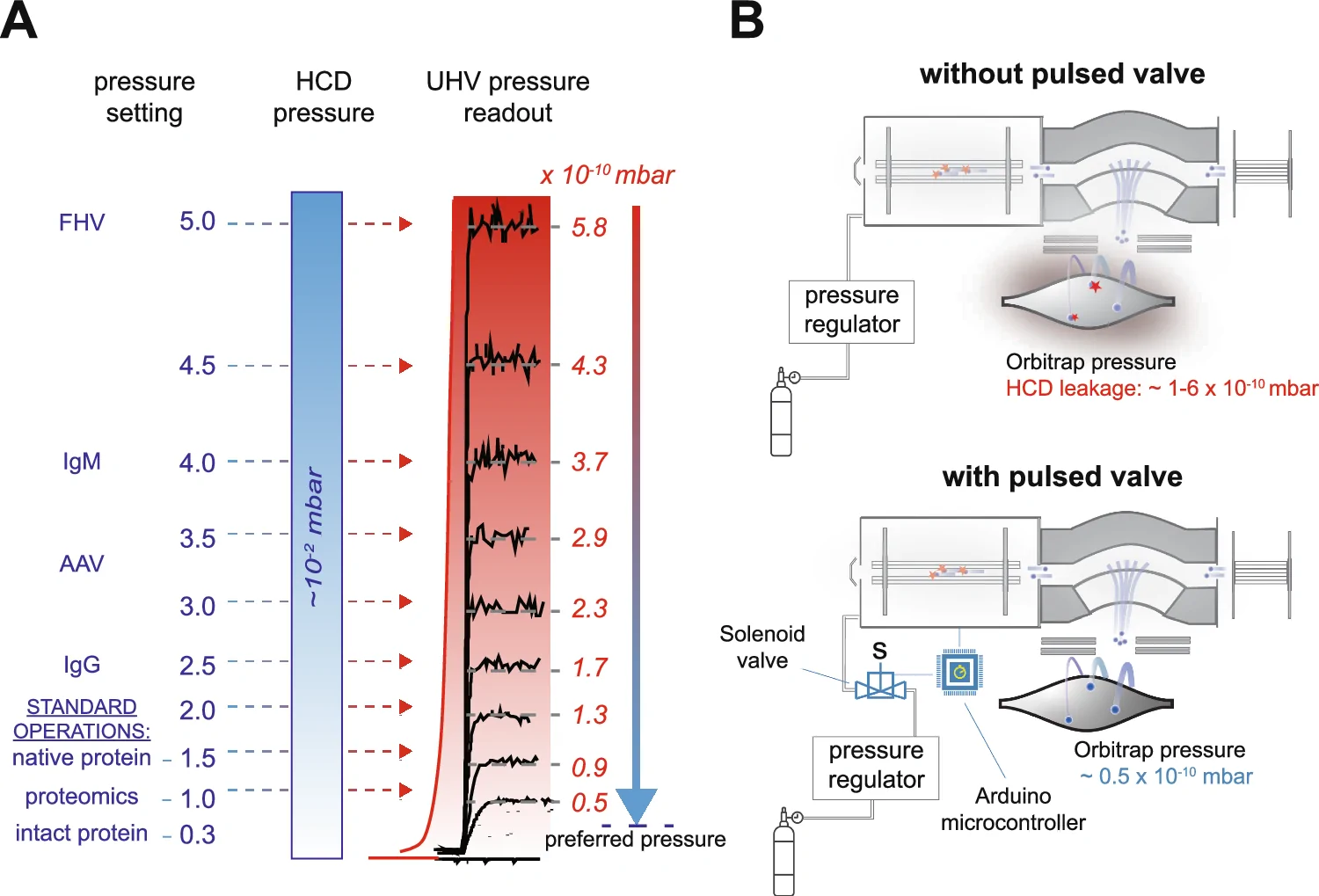
Implementation of a pulsed valve for the timed supply of gas. **A** Larger ions produced by ESI typically require desolvation and collisional cooling before they are optimally transferred and measured in the Orbitrap analyzer. Therefore, different UHMR pressure settings can be applied, with 1.0 typically used for peptides and metabolites and 5.0 for the analysis of very large ions such as those originating from flock-house virus (FHV) particles. Generally, the pressure in the HCD cell reaches in the order of 10^−2 ^mbar. Undesirably, some of the gas leaks into the Orbitrap analyzer, elevating its background pressure. This increases the likelihood that ions will collide with gas molecules during transient recordings. Depicted are the measured pressures in the Orbitrap mass analyzer corresponding to the pressure setting in the HCD cell. The background pressure increases with the HCD pressure setting used, reaching up to 1–6 × 10^−10 ^mbar (multiplied by a factor 2 for the actual Orbitrap mass analyzer pressure, see ref. ^42^). Ideally, for high-resolution mass analysis, the Orbitrap unit is kept at UHV (e.g., < 0.5 × 10^−10 ^mbar). **B** In a conventional Orbitrap-based mass spectrometer, there is continuous gas leakage from the HCD cell into the UHV area (C-trap and Orbitrap analyzer). In the modified setup, an external solenoid valve is placed just before the HCD cell inlet (colored blue). The solenoid valve is operated by an Arduino microcontroller that receives its trigger from the HCD cell. Using this pulsed valve, the desired low Orbitrap pressure < 0.5 × 0^−10 ^mbar can be retained during mass analysis, even when the pressure in the HCD cell is very high.

With the aim of maintaining elevated gas pressures in the HCD cell for ion desolvation and collisional cooling of ions while simultaneously preserving a UHV in the Orbitrap analyzer for mass analysis, we introduced an external solenoid gas valve capable of pulsing with each scan. In the original UHMR design, external gas is introduced at the back of the HCD collision cell for improved ion transmission; however, this gas also leaks into the Orbitrap mass analyzer. This results in a background pressure in the Orbitrap analyzer of about 1–10 × 10^−10 ^mbar (Fig. 1A)^42^. In our design, the additional valve is placed between the pressure regulator and the HCD cell gas inlet (Fig. 1B and Suppl. Fig. 1A). This valve is operated by an Arduino Nano V3 microcontroller that receives a trigger from the HCD cell upon ion ejection at the start of an MS scan (Fig. 1B). To enable ultralong transient scans, each ultralong scan is followed by a shorter, 1-second-long standard scan^38^. Here, we apply 5 of those 1-second-long scans to implement a pulsing scheme that opens the gas valve only during the 5 seconds preceding an ultralong transient scan. This pulsing of the valve at every scan cycle results in a short puff of gas when ions are injected to the HCD cell, with temporary elevation of the gas pressure in the UHV area (~1–6 x 10^−10 ^mbar measured at the Penning gauge), however this is quickly followed by a pressure drop to < 0.5 × 10^−10 ^mbar at the time the ions are trapped and mass analyzed in the Orbitrap analyzer (Suppl. Fig. 1B). In this manner, the Orbitrap gas pressure turns from elevated levels to <0.5 × 10^−10 ^mbar within several seconds. The duration of the gas pulse (seconds) aligns well with that of the long transient scans, which typically last for tens of seconds (Suppl. Fig. 1B).

### Optimized detection and mass analysis of adeno-associated virus particles

**Fig. 2:**
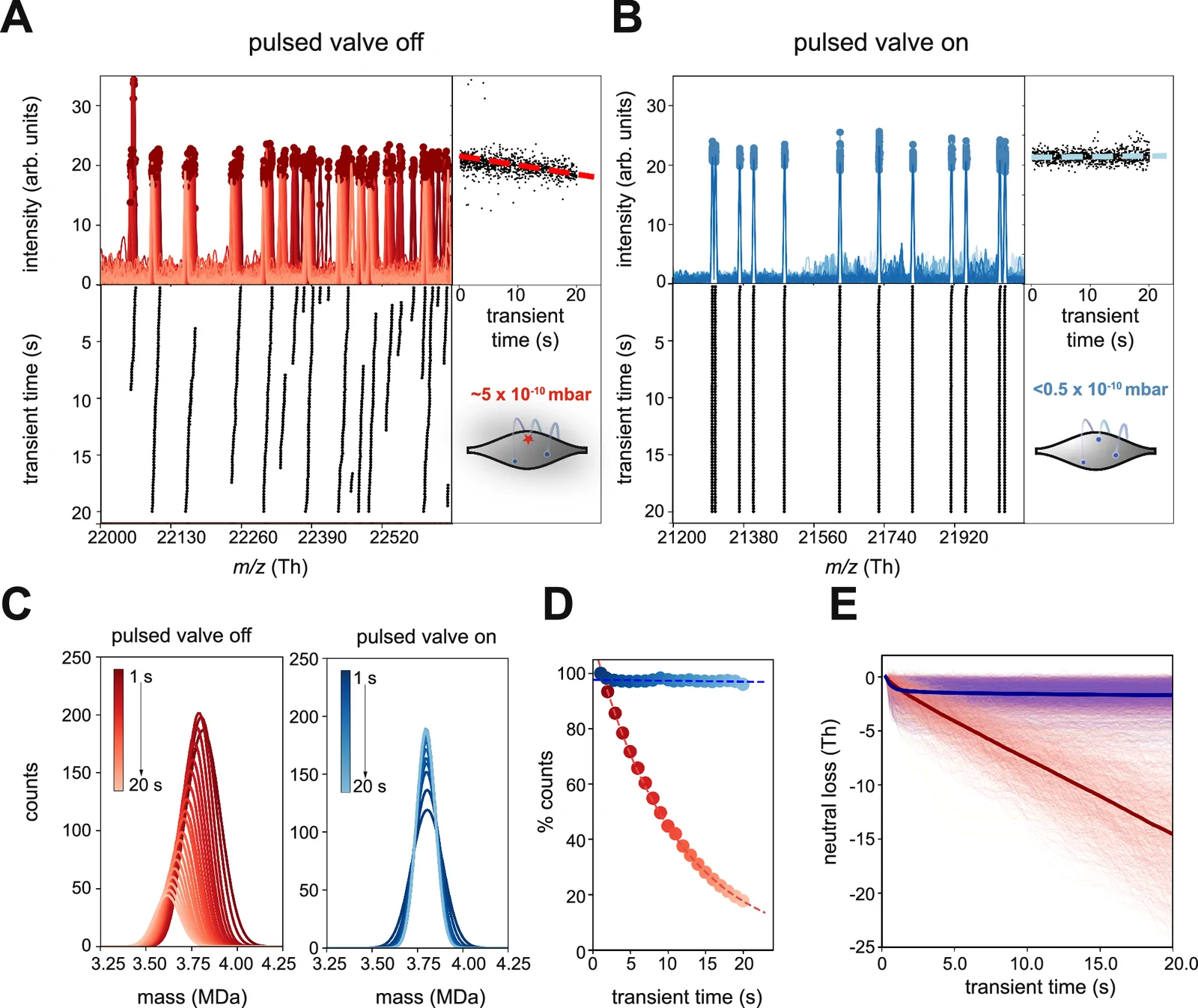
The pulsed valve stabilizes ion trajectories and consequently enhances the detectability of adeno-associated virus particles using charge-detection mass spectrometry. Individual ion signals of rAAVs were monitored in the Orbitrap analyzer during ultralong transient recordings. In all panels, measurements with the pulsed valve off are depicted in red and with the pulsed valve on in blue. **A** Shown are MS spectra with continuous xenon flow and thus high Orbitrap background pressure (~ 5 × 10^−10 ^mbar). Under these standard conditions, with higher background pressure, individual ions undergo extensive neutral losses over time, leading to a steady decline in *m/z* and intensity. **B** Using the pulsed valve, the individual ions maintain their stable *m/z* trajectories and show no neutral or charge losses. In addition, the intensity of each ion remains constant. The MS spectra are segmented into 256-milliseconds time segments, here overlaid from dark to light, with peak centroids indicated by dots. **C** The transient recordings were processed, using incremental timesteps of 1 s, resulting in time-dependent mass histograms (overlaid from dark to light). **D** Focusing solely on stable ions (e.g., no charge losses), the percentage of usable ions is monitored over time. **E** For ions that did not display charge losses, the extent of neutral loss was monitored with continuous background gas or when using the pulsed valve. Signal intensity is shown in arbitrary units (arb. units). Source data are provided as a Source Data file.

To demonstrate the benefits of the pulsed valve for CDMS mass analysis, we first analyzed adeno-associated virus (rAAV) particles as proof-of-concept. Mass analysis of rAAVs has been a key application of CDMS, due to their biopharmaceutical relevance as gene-therapy vehicles^33,43–45^. Here, we focused on empty AAV9 serotype particles, which we previously reported on^41^. To measure these rAAV particles with ultralong transients, we use a frequency-chasing approach that monitors the ion frequency and *m/z*-path at discrete time steps and tracks the particle over the full transient recording^40^. This way, we process the ion over the entire acquisition time without potential perturbations from desolvation or neutral losses. For optimal rAAV desolvation and transmission, a relatively high xenon pressure is used in the HCD cell (pressure setting 3), raising the pressure readout in the Orbitrap analyzer to ~5 × 10^−10 ^mbar (about 2 times the pressure readout compared to using nitrogen at the same pressure setting). This gas pressure promotes optimal desolvation and transmission of the ions but is accompanied by a pronounced effect on the ion stability of the rAAV particles in the Orbitrap analyzer (Fig. 2A). Using the frequency chasing approach, we observe that the rAAV ions experience a substantial drift in *m/z* due to cumulative neutral losses over the duration of trapping. Secondly, ions experience a drag due to consistent collisions with the background gas, resulting in a linear decline in signal intensity. This also has a detrimental effect on mass determination, as signal intensity is directly correlated with charge assignment (Fig. 2C). Lastly, under this relatively higher pressure in the mass analyzer, the rAAV capsid ions often lose one or more charge(s) during the transient recording. This translates into a spontaneous shift in *m/z*, making it virtually impossible to track these ions throughout the full transient recording. Therefore, when sampled over the transient time, rAAV mass determination shows a decline in the number of stable ion particles that can be analyzed, along with a shift in mass and no enhancement in mass resolution as would be anticipated when recording longer transients (Fig. 2C, D).

In sharp contrast, an improvement in all three issues is observed when applying the pulsed valve for short bursts of xenon synchronized with ion injection (Fig. 2B). Neutral losses are constrained, with ions showing straight paths in *m/z* over the full transient time (Fig. 2B, E). Moreover, nearly no charge losses are observed. As a result of the exceptional stability of the ion *m/z* trajectories, we were able to perform a Fourier transform analysis on the entire transient recording, showing virtually no loss of ion signal (with only a few exceptions, mainly due to some neutral loss at the start of the scan) (Suppl. Fig. 2A). In contrast, when the valve is not used, and all other parameters are kept the same, all ion signals are practically lost due to excessive peak splitting (Suppl. Fig. 2B).

The positive effect of applying the pulsed valve on increasing ion stability is also evident in the intensity values, which remain linear over the transient time without the rAAVs experiencing any ion drag (Fig. 2B). Consequently, the artifactual shift in mass observed without using the valve is now eliminated, yielding a more accurate mass of 3.80 ± 0.05 MDa with the pulsed valve on, compared to an erroneous mass of 3.62 ± 0.07 MDa measured with the pulsed valve off (Fig. 2C)^41^. With preservation of the UHV, the sensitivity of CDMS also improves as only a few percent of rAAV ions need to be omitted from mass analysis (with 2959 stable ions at 1 s and 2841 stable ions at 20 s) (Fig. 2D). As an extra bonus, the mass resolution improves when using the valve with a concurrent drop in full-width-at-half-maximum (FWHM), from 204 kDa at 1 s to 122 kDa at 20 s. Overall, implementing and using the pulsed valve leads to clear improvements across all metrics in the mass analysis of rAAV by CDMS.

### Stabilizing ion trajectories of fragile plasmid DNA ions enables their accurate mass analysis

**Fig. 3:**
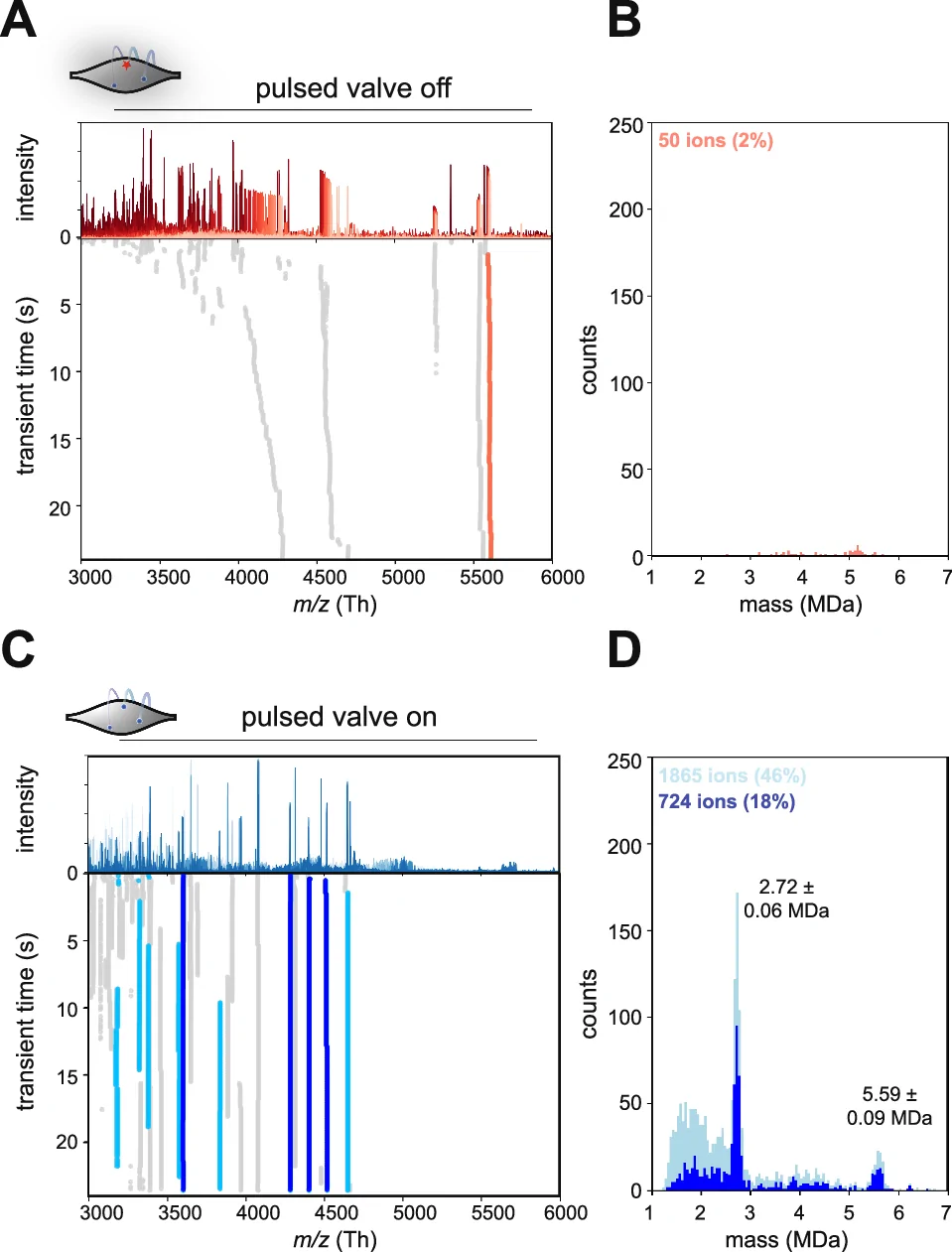
The pulsed valve facilitates ultralong transient recording of fragile ions originating from plasmid DNA. **A** Without the pulsed valve, collisions with background gas have a direct impact on the detectability of ions of plasmid DNA. Shown is an example scan with, on top, the mass spectrum (128-milliseconds segmented transient) and below the ion tracing in *m/z* over the transient time. Due to extensive charge losses, ions appear to move upward in *m/z* (lowering their frequency). Ions that could be traced for a period of at least 12 seconds were selected (*m/z*-path colored red in the bottom panel) and were further processed to extract their mass. **B** Taking together all scans from three independent measurements, only tens of ions (50 out of 3100) were sufficiently stable for at least 12 seconds, and consequently, no accurate mass could be extracted. **C** With the pulsed valve on, substantially more ions remain stable. Ions remaining stable for at least a period of 12 seconds (1865 out of 4054 ions) with their *m/z*-path colored in light blue, and ions that remained stable for at least 22 seconds are depicted in dark blue (724 ions). **D** Taking these selected stable ions, mass peaks could be fitted with a Gaussian function (average mass ± standard deviation indicated) for both the monomer and dimeric plasmid DNA, with masses in good agreement with their expected mass. Source data are provided as a Source Data file.

Like most viral particles, rAAVs are relatively stable in both solution and the gas phase. To probe whether the pulsed valve would also improve the mass analysis of more fragile ions, we analyzed a purely polynucleotide-based sample, namely the 4361-base-pair-long, double-stranded plasmid pBR322. In solution, DNA plasmids have an elongated shape that, during ESI, leads to highly charged, unfolded extended shapes of the particles^26^. It is challenging to desolvate such elongated DNA particles as the negative phosphate backbone retains many counterions (e.g., NH4^+^ solvent)^26,46^. This makes DNA (and RNA) particles vulnerable to collisions in the Orbitrap analyzer, leading to numerous neutral and charged losses, and potentially fragmentation, that can hamper accurate mass analysis^47^. For that reason, plasmid DNA and mRNA constructs could, so far, only be measured using relatively short acquisition times (< 128 milliseconds) (Suppl. Fig. 3)^47^. To investigate whether pulsing the gas flow could also improve the detection and mass analysis of DNA, we applied the ultralong transient recording with the pulsed valve on or the pulsed valve off. With the pulsed valve off, we observed that nearly all pBR322 ions suffered from strong neutral and charge losses, resulting in loss of signal (Fig. 3A). To illustrate, under these conditions, selecting ions that maintained stability for at least a 12-second period (half of the maximum 24-second transient time) retained only a few ions (Fig. 3A, B). Therefore, no mass could be determined under these conditions for pBR322 after 24 seconds of transient recording. When applying the pulsed valve, the prevalence of neutral and/or charge losses from pBR322 ions is much lower and hundreds of stable ions (for a period > 12 s) can be selected (Fig. 3C, D, light blue). In case of instability, it occurs mostly at the start of the scan when the optimal vacuum conditions have not yet been reached. Regardless, when the valve is switched on, a subset of highly stable plasmid particles behaves extraordinarily well, with ions containing a stable period of over 22 seconds (Fig. 3C, D, dark blue). Moreover, the downward drift in intensity values, previously observed for large particles and especially notable here for the highly charged DNA particles, is completely alleviated by using the pulsed valve (Suppl. Fig. 4). These stable ions lead to a sharp mass peak, from which a mass of 2.72 ± 0.06 MDa could be determined, close to the theoretical mass of 2.69 MDa. Also, particles are detected having double this mass (5.59 ± 0.09 MDa), indicating the presence of pBR322 dimers. Previous studies showed that DNA plasmids are prone to shearing during electrospray, a feature we also observe here with plasmid fragment ions detected close to ~1.8 MDa^26^. Focusing on the intact pBR322 plasmids, they appear enriched within the highly stable ion subset (*m/z*-path > 22 s): 303 intact plasmids out of 724 highly stable ions versus only 544 intact plasmids out of 1865 total measured ions (Fig. 3D). This suggests that the sheared plasmid fragments are less stable than the intact plasmids. In agreement with this observation, plasmid fragments are completely absent in the mass histogram when the pulsed valve is off (Fig. 3B). From this data we conclude that we can trap, detect and mass analyze highly fragile DNA plasmid ions using transients up to 10-20 seconds, but exclusively when using the pulsed valve, which opens avenues to mass analyze other fragile analytes such as mRNA or ssDNA constructs.

### Charge-detection mass spectrometry of immunoglobulins using the pulsed valve

**Fig. 4:**
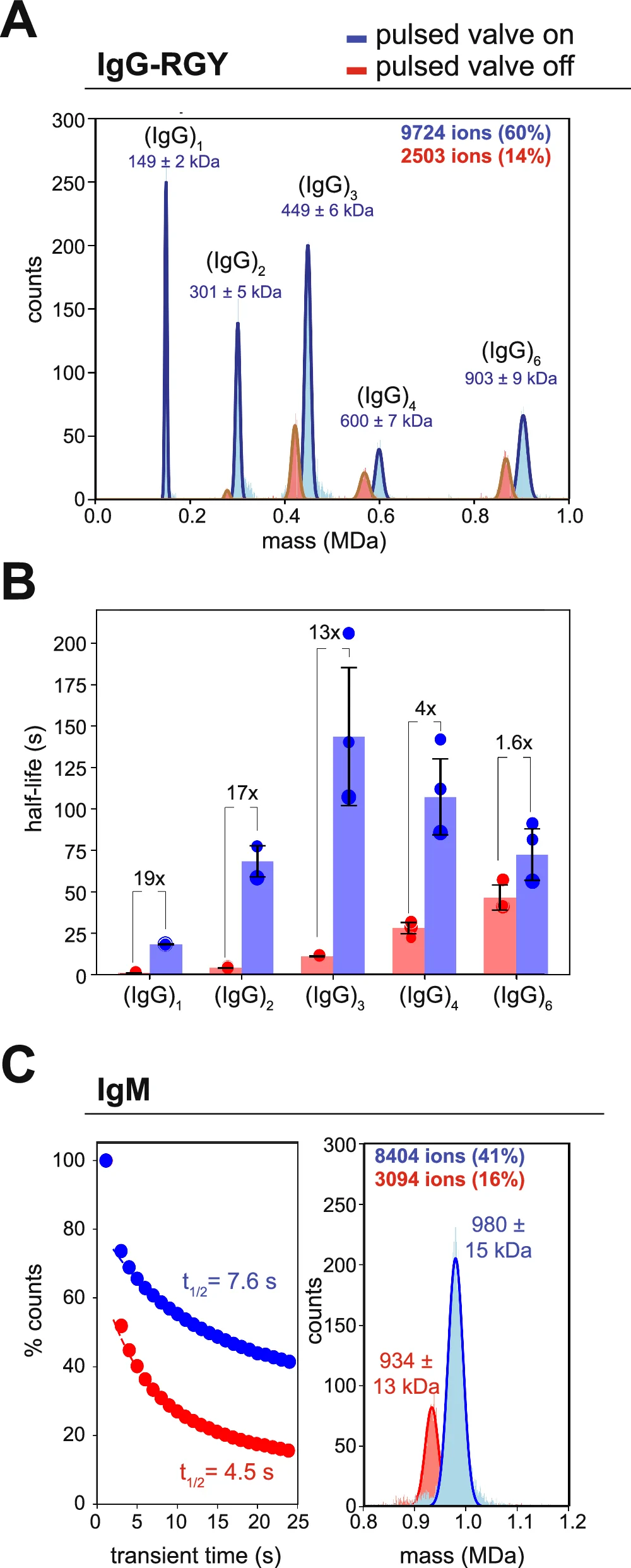
CDMS of immunoglobulin oligomers. **A** Ions from the IgG1-RGY oligomers were analyzed either without or with the pulsed valve on (resp. in red and blue). Without the pulsed valve, all oligomers are affected, but especially the smaller monomeric and dimeric species. With the pulsed valve on, more ions remain stable, including those from the monomers, resulting in mass histograms for all IgG1-RGY oligomers. Indicated are the average mass ± standard deviation from the Gaussian fits from the masses with pulsed valve on. **B** We defined effective ion population half-life times (t_½_) of the exponential decay phase under conditions without (red) or with the pulsed valve on (blue). The half-life times were generated from three independent repeats, with error bars indicating the standard deviation and the center of the error bar representing the mean. Each dot represents a measurement, with the dot size related to the total number of ions measured. **C** CDMS of IgM pentamer. Shown is the ion survival (left) and mass histogram (right) of IgM measured without and with the pulsed valve on (two independent repeats, which accumulated in a total of ~20,000 starting ions for both conditions). Without a pulsed valve, only 16% of these ions retain stable trajectories, while with a pulsed valve, this extends to 41%. Consequently, the calculated half-life times reveal about a 1.6-fold increase in ion stability. Moreover, only by using the pulsed valve can a correct IgM-J mass be measured. The mass histogram is generated after processing the full 24-second transient. Indicated are the average mass ± standard deviation from the Gaussian fits. Source data are provided as a Source Data file.

We next directed our attention to antibody-based biopharmaceuticals. We focused on a range of both low- and high-molecular-weight immunoglobulins (150–1000 kDa). We first evaluated the pulsed valve’s performance by analyzing an IgG1-RGY triple mutant using CDMS, IgG1-RGY is known to form oligomers in solution, especially hexamers^48^. Without using the pulsed valve, extended trapping of these IgG1-RGY oligomers in the Orbitrap analyzer nicely displayed the biased effect of background gas collisions across different-sized oligomers (Fig. 4A, in red). As expected, ions originating from relatively lower-mass ions (i.e., IgG monomer and dimer) were found to be highly unstable under relatively high Orbitrap background pressure. In fact, ions of IgG monomers and dimers exhibited excessive neutral losses or even disappeared completely during the measurement, implying that no useful mass information could be obtained for IgG1 monomers already early on during the measurement (Fig. 4A and Suppl. Figs. 5, 6A and 6B). To illustrate these effects further for each oligomer, we defined effective ion population half-life times (t_½_) by fitting an exponential decay function to the decay phase (here, ≥3 seconds at least 4 × 512-milliseconds segments). We calculated these t_½_‘s for each of the IgG oligomers under conditions with or without the pulsed valve on (Fig. 4B and Suppl. Figs. 6A, C). Using the pulsed valve, we observed a dramatic improvement across all oligomeric variants (Fig. 4A, in blue), especially with the stability of IgG1 monomer ions improving sharply, extending t_½_ by a factor of 19 (Fig. 4B). Also, for the other oligomers the stability of the ion trajectories was enhanced with the pulsed valve on, although as expected the fold-change gradually diminished with larger oligomer size (Fig. 4B). When measuring this broad range of masses, the t_½_ for bigger oligomers appears to diminish slightly, because they are less well desolvated and can experience some neutral loss. Exclusively, using the pulsed valve, we were successful in recording long transients for both the IgG1-RGY hexamer and IgG1-RGY monomer ions, obtaining an accurate and expected monomer mass of 149 ± 2 kDa (Fig. 4A).

Next, we also analyzed a recombinant IgM (i.e., J-chain coupled pentamer, ~980 kDa). The IgM pentamer is decorated with ~50 glycosylation sites that can easily fragment during mass analysis, requiring a delicate balance between optimal desolvation (high pressure in the HCD cell) and stable detection (low pressure in the Orbitrap analyzer)^28,49^. As seen for the IgG-RGY, also IgM benefited greatly from using the pulsed valve (Fig. 4C). Without a pulsed valve, many ions cannot be used for mass analysis due to strong desolvation and neutral losses. Moreover, ions experience an intensity drift due to continuous collisions with background gas, causing an artefactual decrease in mass (Fig. 4C and Suppl. Fig. 5B). In comparison, with the pulsed valve on, more IgM ions remain stable over the 24-second mass analysis in the Orbitrap mass analyzer, rendering an overall mass of 980 ± 15 kDa in line with the expected mass. Because of the excellent stability during mass analysis with the pulsed valve on, we also monitored the ion resolution of each individual ion over the transient time (Suppl. Fig. 7). As with frequency chasing (solely based on *m/z*), ion resolution and ion intensity can be used as proxies for measurement quality^50^. Using ion resolution and intensity, the results clearly showed the benefit of a pulsed nitrogen source, achieving a very high resolution (R > 400,000), indicating that these IgM ions are stable for long periods of time.

### Antibody-protein A binding examined by charge-detection mass spectrometry

**Fig. 5:**
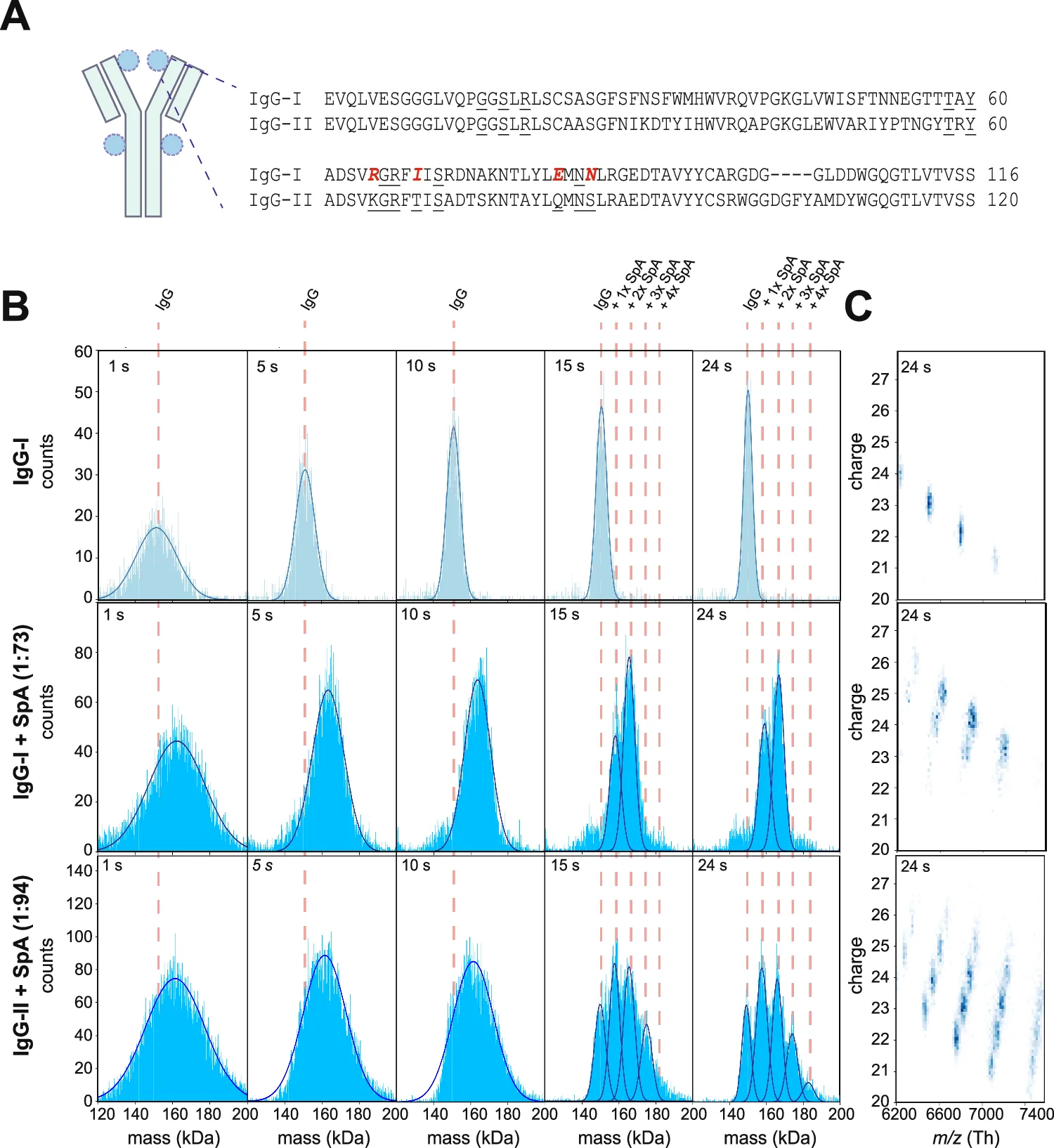
Using the pulsed valve to resolve antibody-SpA complexes by CDMS. **A** SpA-B interaction was studied when binding to IgG-4497 (IgG-I) and Trastuzumab (IgG-II). Shown are the distinctive VH3 amino-acid sequences with the residues expected to interact with SpA underlined. Residues in red diverge from the suspected binding sequence (see ref 51). **B** Analysis of SpA-B binding to IgG-I and IgG-II. IgG-I, in the absence of SpA-B, was analysed by CDMS using the pulsed valve, which showed a single mass of ~150 kDa (light blue). When IgG-I was pre-incubated with excess SpA-B, we observed maximally the binding of two copies of SpA-B (approximately separated by 8.4 kDa), but for IgG-II, we observed the binding of maximally 4 copies of SpA-B (dark blue). Only when using transient recording above 15 s can the binding of SpA-B be well resolved. The number of inferred SpA-B domains binding to IgG is indicated by a red dashed line. IgG and SpA molar ratios are indicated on the left. **C** Depicted are the 2D-histograms of charge versus *m/z* of ions corresponding to the full 24-second transient measurement in panel B on the left. Source data are provided as a Source Data file.

Next, we probed whether the enhanced CDMS acquisition strategy provides sufficient mass resolution to investigate the binding of smaller protein domains to IgGs. We analyzed the binding of a small protein, namely the ~8.3 kDa B-domain of the *Staphylococcus aureus* protein A (SpA-B). SpA-B is known to bind well to IgG, with one copy binding to each Fc chain in the antibody, but it can also interact with the Fab domain. SpA binds the Fab region when the antibody heavy chain variable domain belongs to the human VH3 clan and contains a specific arrangement of framework/CDR residues that create a so-called B cell superantigen site. Not all VH3 sequences bind equally well, and non-VH3 antibodies essentially lack Fab binding (Fig. 5A)^51,52^. We here assessed two IgG mAb variants, namely IgG-4497 (IgG-I)^53^ and Trastuzumab (IgG-II), as we hypothesized that they could display different binding stoichiometries based on their VH3 sequences, from which we reasoned that they could bind two or four SpA-B domains. Measuring IgG-I by CDMS in the absence of SpA-B resulted, as expected, in a single peak at 150.1 ± 2.4 kDa (Fig. 5B). Following incubation with SpA-B at high excess, we could confirm that this IgG does bind maximally only 2 SpA-B domains, indicated by two mass peaks shifted by ~9 kDa and an additional ~8 kDa, compared to the SpA-free IgG-I (Fig. 5B). Performing CDMS analysis at different transient recording times, we could already observe clear mass shifts using a transient of 5 seconds. However, only with a 24-second transient can the binding of 1 and 2 SpA-B copies be well mass-resolved, as also became evident from the charge distribution of the ions (Fig. 5B, C). For IgG-II, incubation with high excess of SpA-B generated masses indicating the binding of 3 and possibly 4 SpA-B domains (Fig. 5B and Suppl. Fig. 8). Again, only by using longer transients could the number of bound SpA-B domains be well resolved. To be able to record IgG’s up to 24 seconds, the pulsed valve turned out to be vital (Fig. 4A and Suppl. Figs. 5A and 6), which also proved key here to elucidate the stoichiometry of SpA-B binding (Fig. 5B). Altogether, this demonstrates that the pulsed valve improves ion stability in the mass analyzer up to a point where we can accurately measure IgGs and binding of small domains or epitopes of just a few kDa.

## Discussion

Ion detection in an Orbitrap analyzer occurs by recording the image current originating from the ion’s charge oscillating along the axis of a central spindle-like electrode in an electrostatic field^54,55^. By applying a Fourier transform to this image current, the frequency of the ion’s oscillations can be determined (interrelated to the ion *m/z*), resulting in a mass spectrum. For Orbitrap-based CDMS, not only the frequency but also the image current amplitude is important, as it scales with the ion’s charge^37^. The longer the image current of the oscillating ion can be measured, the more accurately the frequency (*m/z*) and charge, and thus the particle mass, can be assessed^55^. This is one of the main reasons the Orbitrap analyzer is embedded in a UHV system, as collisions with residual gas molecules will negatively affect ions’ trajectories and thus the resulting image current and mass spectra. However, for optimal ion transmission, other compartments such as the ion source, quadrupole mass filters, and the HCD cell might require elevated pressures. This is especially true for the HCD cell, where elevated gas pressure is used to effect collision-induced dissociation to obtain MS^2^ fragments, improve the desolvation after ESI, and/or collisionally cool ions to improve their transmission efficiency to the mass analyzer^13,56,57^. Unfortunately, the externally supplied gas in the HCD cell leads to an undesired increase in the Orbitrap pressure despite multiple stages of differential pumping^42^. Here, we found a solution to resolve the undesirable elevated background pressure in the Orbitrap analyzer in the form of a pulsed gas valve that only temporarily introduces gas for ion desolvation and collisional cooling into the HCD cell and C-trap. After a pulsed elevated pressure is achieved in the HCD cell, the valve is closed, and the residual gas is pumped away (Fig. 1B and Suppl. Fig. 1B). This way, we improve transmission and desolvation of a broad range of analytes to the Orbitrap mass analyzer while keeping the analyzer at UHV during the transient recording. We demonstrate that ions can oscillate stably with improved detection of their frequency and charge (Figs. 2B, 3C). In this modified UHMR mass spectrometer, the addition of the pulsed valve combines optimal transmission of a broad range of particles (rAAVs, DNA plasmids, IgM, and IgG) with the stable recording of their transient signals in the Orbitrap mass analyzer, even up to ~24 seconds (Figs. 2–4).

In protein-centric, native MS applications (e.g., ensemble native MS, native top-down, CDMS), analytes typically have higher masses and lower charge states, therefore, ions travel more slowly through the front end of the mass analyzer, and their trajectories are more prone to becoming off-track, decreasing their transmission efficiency. Especially for those analytes, an elevated HCD pressure has been proven essential, both to optimally desolvate ions (removing residual water and small molecule adducts) and to collisionally cool them. When applying an ultralong transient recording, by coupling an external data system to the UHMR mass spectrometer, the long acquisition times demonstrated it can improve precision of the single-molecule CDMS but it also clearly revealed the instability of individual ion trajectories when trapped for longer times, as they can cover trajectories of over hundreds of kilometers, increasing the chance of colliding with background gas molecules (Figs. 2A, 3A)^38,41,50,58^. These collisions are detrimental to most ions, especially for fragile analytes such as RNA and DNA molecules and protein assemblies that carry labile modifications, such as phosphorylation and glycosylation (Figs. 3 and 4)^23,28,47,58^. Also, smaller ions, with lower *m/z* and higher frequency, generally experience more collisions (despite a higher mean free path) due to the greater distance they travel^40,41^. Moreover, they have a lower capacity to absorb collisional energy (smaller collisional cross section, higher transfer of center-of-mass energy). For such ions, a reduction in collisions with the background gas would be preferred. We initially aimed to address these detrimental collisions by reducing the charge state of the analytes, as lower charges (higher *m/z*) lead to lower frequencies and, consequently, fewer collisions with lower collision energies, and showed that this can help several analytes^41,50^. However, by using the pulsed valve instead, the CDMS analysis of both high and low *m/z* analytes can be substantially improved as fewer collisions lead to less loss of ions, and therefore we can record long transients of a substantially higher number of individual ions, improving the accuracy in charge detection, signal-to-noise, and mass accuracy (Figs. 2–4). Moreover, by rapidly removing the background gas from the Orbitrap analyzer, the drag experienced by ions is eliminated, keeping their intensities stable and avoiding potential artifacts in charge and mass determination (Fig. 2B and Suppl. Figs. 4C and 6D).

In standard Orbitrap operation mode, such as in proteomics and metabolomics experiments, the ion trapping and recording times are relatively short (16–128 milliseconds for high-throughput proteomics and MS/MS scans, 256–1024 milliseconds for high-resolution MS¹ scans). Under such conditions, already a significant fraction of the ions do not survive the full transient recording time; however, this does not dramatically affect the measurements, as in ensemble measurements, millions of ions are typically recorded simultaneously, so sufficient signal is still obtained. Nonetheless, the rapid decay of the transient signal in ensemble mode leads to reduced ion signal and lower attainable mass resolution. With extended ultralong transient recording, we can improve the resolution. Evidently, this procedure, whereby we aim to record ~24 s transients, slows the entire mass measurement duty cycle, but in our case, using the pulsed valve, the additional time is worthwhile, as it results in massive improvements in the stability of the ions’ trajectories within the Orbitrap analyzer. We show, for instance, that under standard pressure conditions, nearly no ions from an IgG1 antibody survive the ~24 s of Orbitrap trapping, making it impossible to determine their mass by CDMS (Fig. 4A). In contrast, employing the pulsed valve, substantial amounts of IgG1 ions do survive the full transient, allowing accurate determination of their charge and *m/z*, and thus mass (Figs. 4A, 5B). Recording longer transients improves mass resolving power, which we here demonstrate by observing resolved ion signals from an IgG antibody (~150 kDa) bound to several copies of a~8.3 kDa binding partner (SpA-B), but also this could only be achieved by using the pulsed valve to retain UHV conditions in the Orbitrap analyzer during the transient recording (Fig. 5). Even at shorter transient times (3-6 seconds), which can be recorded on a standard UHMR mass spectrometer without a FTMS Booster, the pulsed valve improves CDMS measurements, showing stable mass accuracy and ion accumulation for both larger (IgM) and smaller (IgG1-RGY) immunoglobulin complexes (Suppl. Fig. 5).

In conclusion, the implementation of a pulsed valve, used to regulate the temporal elevation of gas-pressure in the HCD cell and mass analyzer, improves the sensitivity, charge and mass accuracy, and resolving power for analysis in the Orbitrap analyzer. Although demonstrated here, particularly for measuring larger assemblies (e.g., plasmid DNA, IgM, and AAV particles) in CDMS applications, maintaining UHV in the Orbitrap mass analyzer is potentially beneficial for all mass analysis.

## Methods

### Samples

The empty rAAV9 serotype sample was kindly provided by M. Thomann (Roche, Penzberg, Germany). The IgG1-RGY was provided by the team of J. Schuurman (Genmab, Utrecht, Netherlands). Recombinant anti-Strep IgM, IgG-I (IgG-4497) and SpA-B were prepared by the group of S.H.M. Rooijakkers (University Medical Center Utrecht, Utrecht, Netherlands). Roche provided IgG-II (Trastuzumab). We purchased the pBR322 plasmid from Thermo Fisher Scientific (Vilnius, Lithuania). The different protein-based samples were buffer exchanged to ammonium acetate solutions (ranging between 50-150 mM) using either Micro Bio-Spin columns from Biorad (i.e., rAAV), Amicon Ultra Centrifugal filters from Millipore (i.e., IgM) or Zeba 7 K MWCO spin columns from Thermo Fisher Scientific (i.e., IgG-I, IgG-II and SpA-B) according to the manufacturer’s instructions. We obtained IgG and SpA-B complexes (following buffer exchange to 150 mM ammonium acetate) by incubating 0.7 μM IgG at room temperature for 1 h with 50 and 70 μM SpA-B, respectively, for IgG-I and IgG-II. Plasmid DNA, pBR322, was initially buffer exchanged to 40 mM ammonium acetate using an Amicon Ultra Centrifugal filter according to the manufacturer’s instructions. A final washing step of the pBR322 in the filter was performed with H_2_O to retain pBR322 in an estimated 0.2–0.4 mM ammonium acetate solution as described by Schultz et al^59^. For all buffer-exchanged samples, we further diluted the samples with ammonium acetate to measure them in the single-ion regime when needed. For nano-ESI, a few μL of the (diluted) samples were loaded into in-house pulled, gold-coated borosilicate capillaries.

### Ultralong transient charge detection mass spectrometry

The ultralong transient CDMS measurements were performed on a modified Thermo Scientific Q Exactive UHMR Orbitrap mass spectrometer coupled to an FTMS Booster X2 (Spectroswiss, Lausanne, Switzerland) as described before^38^. A solenoid valve (Burkert 6604) was placed in close proximity to the HCD cell after the collision gas valve. For each sample, we optimized transmission and desolvation according to the settings given in Supplementary Table 1. Gas pressures were regulated by adjusting the pressure setting and reading out the pressure using the Penning gauge. Importantly, this readout value is specific for each UHMR instrument. Ultralong transient measurements were done following a scan schedule where each long transient scan ( > 20 s) was followed by 5 consecutive buffer scans of 1 s. The buffer scans were not used for further processing but only for stabilizing the electronic system. In general, the pulsed valve (when used) was opened during the buffer scans for about 4.75 seconds and closed just prior to the long scan, remaining closed for the duration of the ultralong transient scan ( > 20 s). The Arduino Nano microcontroller received its trigger from the HCD cell when ions are pushed out by a steep voltage drop. Once this HCD trigger associated with the long transient scan is detected, the system keeps the solenoid valve closed for a defined delay time, then opens it using a short high-power pulse followed by a lower holding signal for the set valve time. Once this delay-valve sequence is completed, the valve closes again, and the next pulse cycle starts at the following trigger. We measured the samples with the valve pulsing (pulsed valve on) or continuously open (pulsed valve off) for about 45–90 min. Only for the pBR322 plasmid did we alternate between long scans with the valve open and closed within a single measurement. The open- and closed-valve scans were then processed separately. Samples were measured once unless stated otherwise in the figure legends.

### Data processing

The raw signals from the FTMS Booster X2 recordings were processed using in-house developed Python (version 3.12.1) scripts as described earlier and available in the exemplary code^38,41^. In short, following initial Peak-by-Peak processing (Spectroswiss version 2024.05.0), the transient files were analyzed using a frequency chasing approach, with time segments converted to magnitude FTs (mFT) using three times zero-filling and apodization with a full-window Hamming function^40^. The resulting frequencies (in *m/z*) were monitored over time. Ion signal intensity to charge conversion was done based on a calibration with GroEL as reported previously^38^. Unstable ion trajectories were removed if a rolling standard deviation of the last four segment *m/z* values exceeded a sample-specific threshold (Supplementary Table 1). From the ions that remained stable, mass histograms were constructed with appropriate bin sizes for the mass range. The average mass ± standard deviation was determined from a Gaussian fit of the mass histogram. Ion counts were monitored over the transient time by setting the ion counts to 100% at 1 s, then applying 1-second incremental timesteps. For IgG-RGY and IgM, the ions were counted from 3 s onwards to account for the rolling standard deviation, and at least four consecutive 512-milliseconds segments. The decrease in ion counts was fitted to a single-exponential decay. For IgM, the single-exponential decay fit included a baseline value.

For the rAAVs and IgM, we also applied a non-segmented, complete mFT as shown in the supplementary data. The increase in resolution can be used as a proxy for ion stability, as only a stable frequency (in *m/z*) will enhance resolution. For IgM, we calculated the ion peak resolution over the transient time by fitting a Gaussian to the peak and obtaining the FWHM (Suppl. Fig. 7A). Because the true intensity value corresponding to the ion charge is reached only when the ion remains stable throughout the entire transient, we applied a filter step based on both the ion intensity and resolution to extract stable ions.

For the pBR322 plasmid DNA, we noticed that ions display stretches of stability in time, but not necessarily from the start of the ultralong transient. Therefore, we applied a stability criterion in which ions are required to be steady in *m/z* for at least 12 seconds out of the 24-second transient. This way, ions do not necessarily have to be stable from the start of the scan, while at the same time, we avoid accidentally counting ions twice. To enrich for the highly stable ions, we also applied a 22-second stability requirement.

### Reporting summary

Further information on research design is available in the Nature Portfolio Reporting Summary linked to this article.

## Supplementary information

Supplementary Information

Reporting Summary

Transparent Peer Review file

## Source data

Source Data

## Data Availability

Data supporting the findings of this study are available within the paper and its Supplementary Information. Source data are provided with this paper. Alternatively, the data is available from the corresponding author upon request. An example dataset is provided alongside the example code. Source data are provided with this paper.

## Code availability

An example Python script for long transient data processing is available in the Zenodo database under https://doi.org/10.5281/zenodo.20690164.
