# Supplementary material for: Modulating pressure in the Orbitrap improves sensitivity and mass resolution in charge detection mass spectrometry
Source: Nat Commun. 2026 Aug 13;17:9690. doi: 10.1038/s41467-026-76691-1 (PMC13558686; doi:10.1038/s41467-026-76691-1)
Supplement: Supplementary file 1 [file 41467_2026_76691_MOESM1_ESM.pdf]

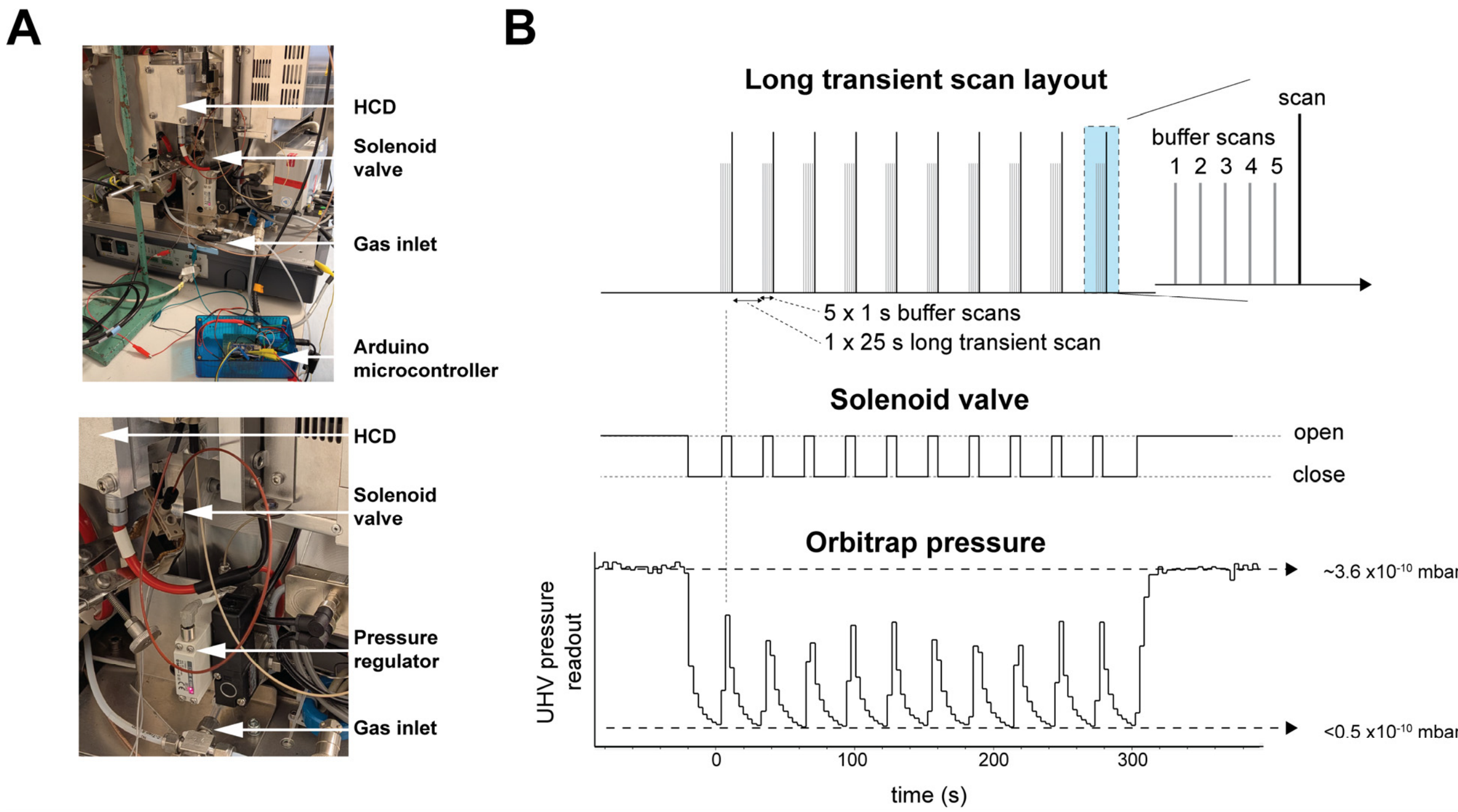

Supplementary Figure 1: Timed injection of gas into the HCD cell by using the pulsed valve. A) Images of the modified Q Exactive UHMR Orbitrap mass spectrometer show the different components and the newly installed solenoid valve. **B)** Top: The scan layout for ultralong transient measurements consists of 5 × 1-second buffer scans followed by the recording of the actual ultralong transients of 25 seconds. Middle: During buffer scans, the pulsed valve is opened, allowing gas to flow into the HCD cell. During an ultralong scan, the valve remains closed for the full 25 seconds. Bottom: Due to the pulsing of the valve, a short puff of gas (nitrogen or xenon) is supplied to the HCD cell. This pressure can be measured with a Penning gauge near the Orbitrap analyzer, which detects residual gas leakage from the HCD cell into the Orbitrap region. Only during the buffer scans is a short, sharp increase in gas detected. Because the Penning gauge reads the pressure only every few seconds, the height of this gas peak appears variable. Following the gas peak, an immediate, strong reduction in pressure is visible, followed by a longer phase of further reduction. While the pressure continues to decrease, ion stability can already improve from the onset of the scan.

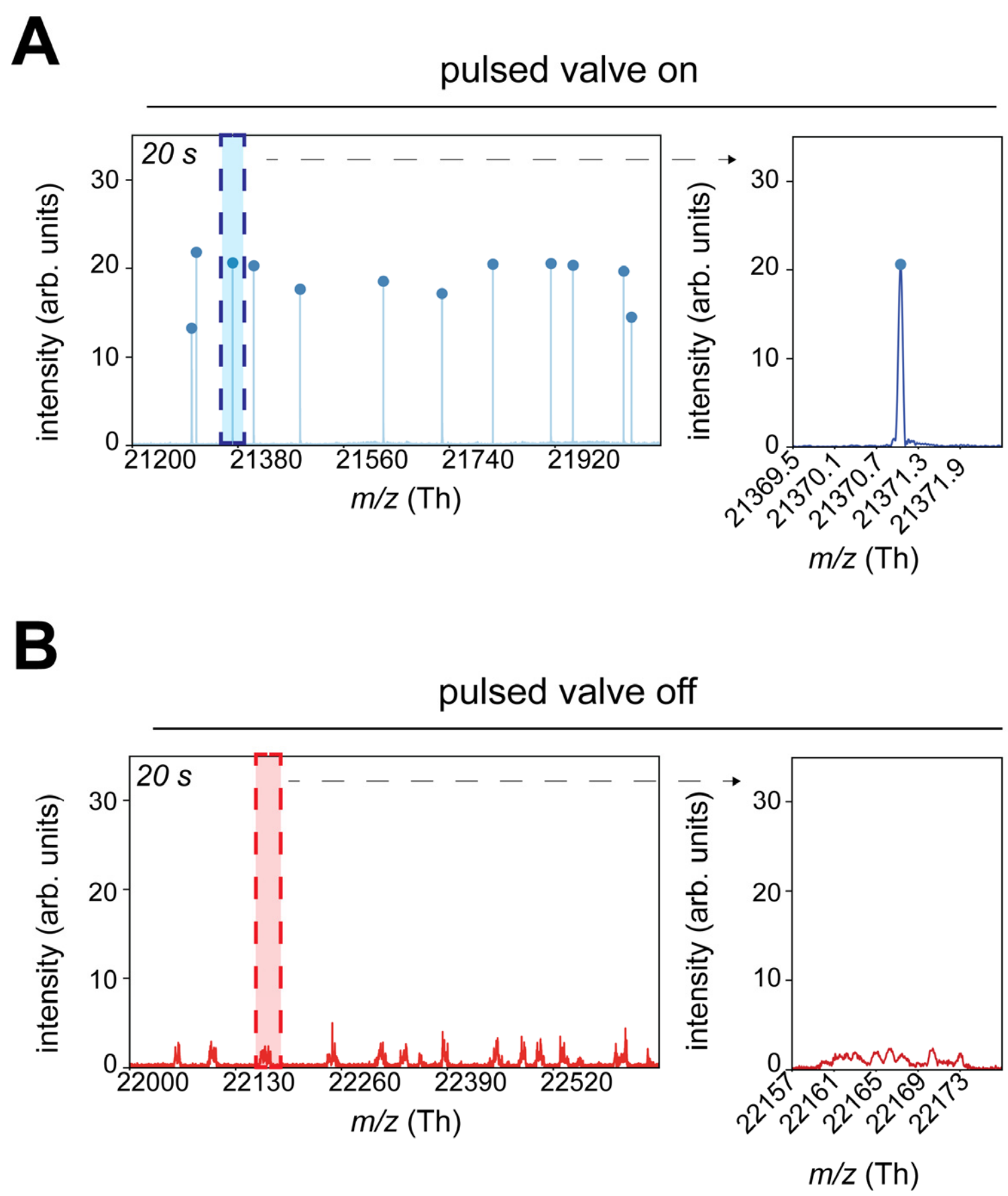

Supplementary Figure 2: Analysis of rAAV individual ions using unsegmented 20-second transients. In contrast to frequency chasing, the transient can also be analyzed in its entirety at once (unsegmented). **A)** With the pulsed valve on, this showed that the rAAV individual ions do not drift anymore in frequency, yielding high-resolution single-ion signals for each ion. A close-up of a selected individual ion is shown on the right, highlighting the improvement in intensity, resolution, enabling accurate mass analysis. **B)** With the pulsed valve off, the gas leakage to the Orbitrap analyzer causes ions to collide with the background xenon gas. This causes the ions to drift in frequency, resulting in severe peak splitting. A close-up of the selected individual ion is shown on the right, depicting that these collisions have a detrimental effect on intensity, resolution, and thus accurate mass analysis. Signal intensity is shown in arbitrary units (arb. units). Depicted signals in blue, pulsed valve is on; in red, pulsed valve is off.

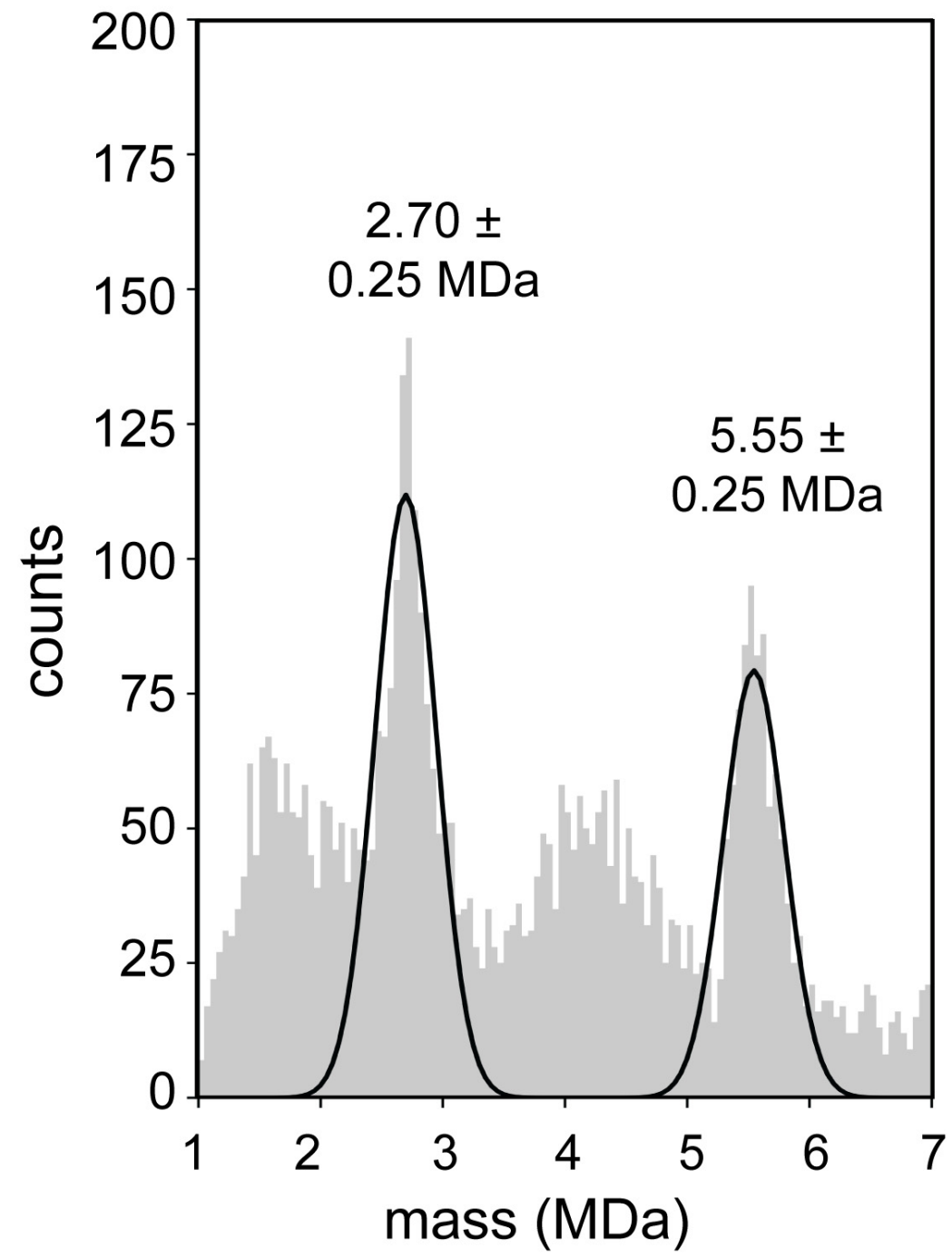

Supplementary Figure 3: Charge-detection mass spectrometry measurement of pBR322 applying a short transient. The plasmid DNA vector pBR322 was measured by applying a conventional CDMS method with the pulsed valve off and a shorter transient length of 64 milliseconds. The DNA ions remain stable during this short transient length, so a monomeric and a dimeric peak can be detected at 2.70 and 5.55 MDa, respectively. Due to the shearing of the DNA during ESI ionization, a high level of background can also be observed. The background ions hinder the proper fitting of a Gaussian curve to the pBR322 peaks.

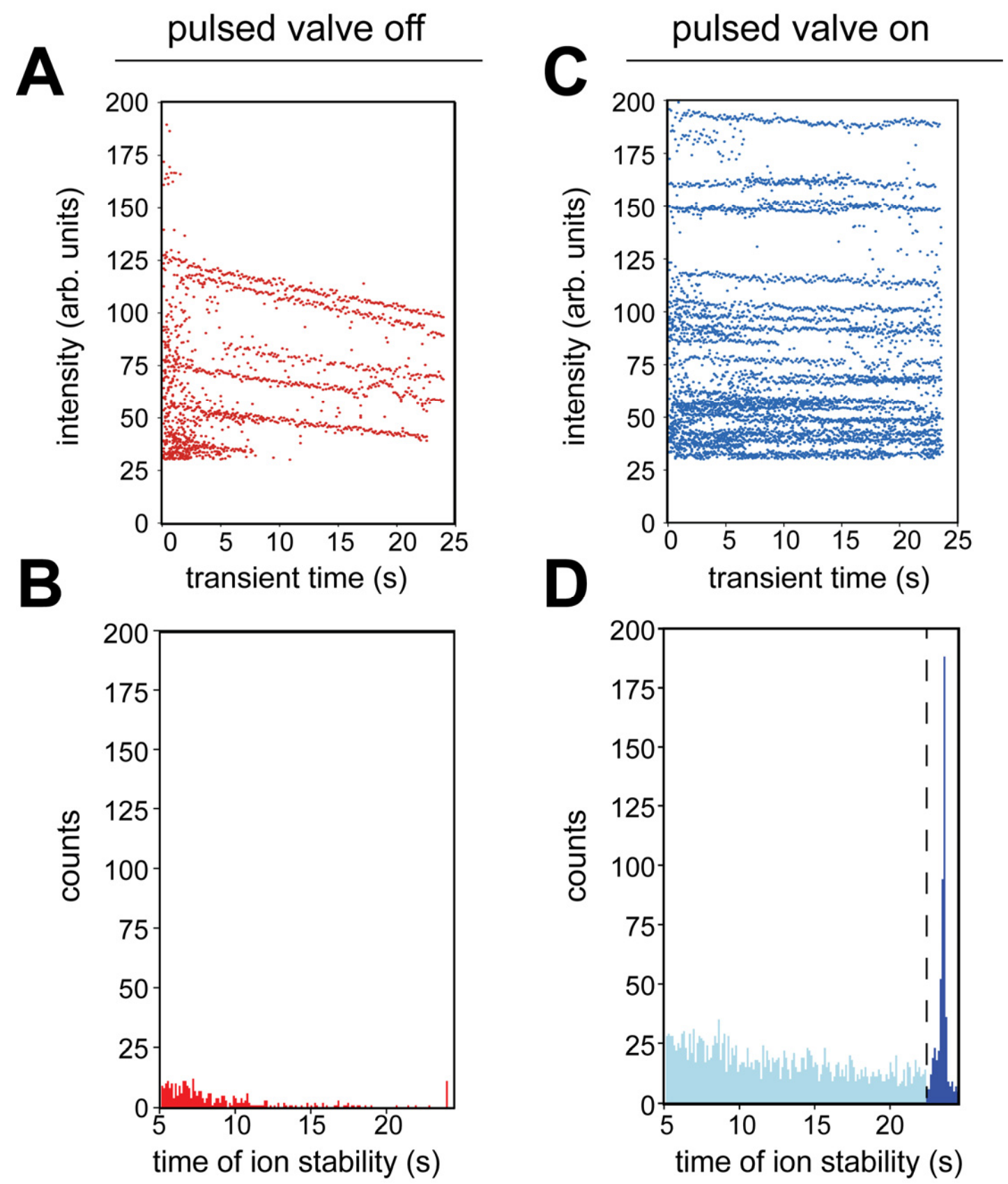

Supplementary Figure 4: Stability analysis of individual plasmid DNA ions without and with the pulsed valve. A) Without restriction of residual gas flow into the Orbitrap analyzer, the individual ions of DNA plasmids experienced a strong ion drag (in red), which resulted in a linear decrease of their intensity values. **B)** Moreover, the time that ions remain stable in *m/z* revealed that nearly none of the individual ion holds its oscillation frequency for the full 24-second transient. **C)** With the pulsed valve on (in blue), the ion intensity values remained rather constant, and **D)** the time that plasmid ions remained stable in *m/z* was also much improved (light blue), with a substantial population of ions that were stable for over 22 seconds in the Orbitrap mass analyzer (dark blue).

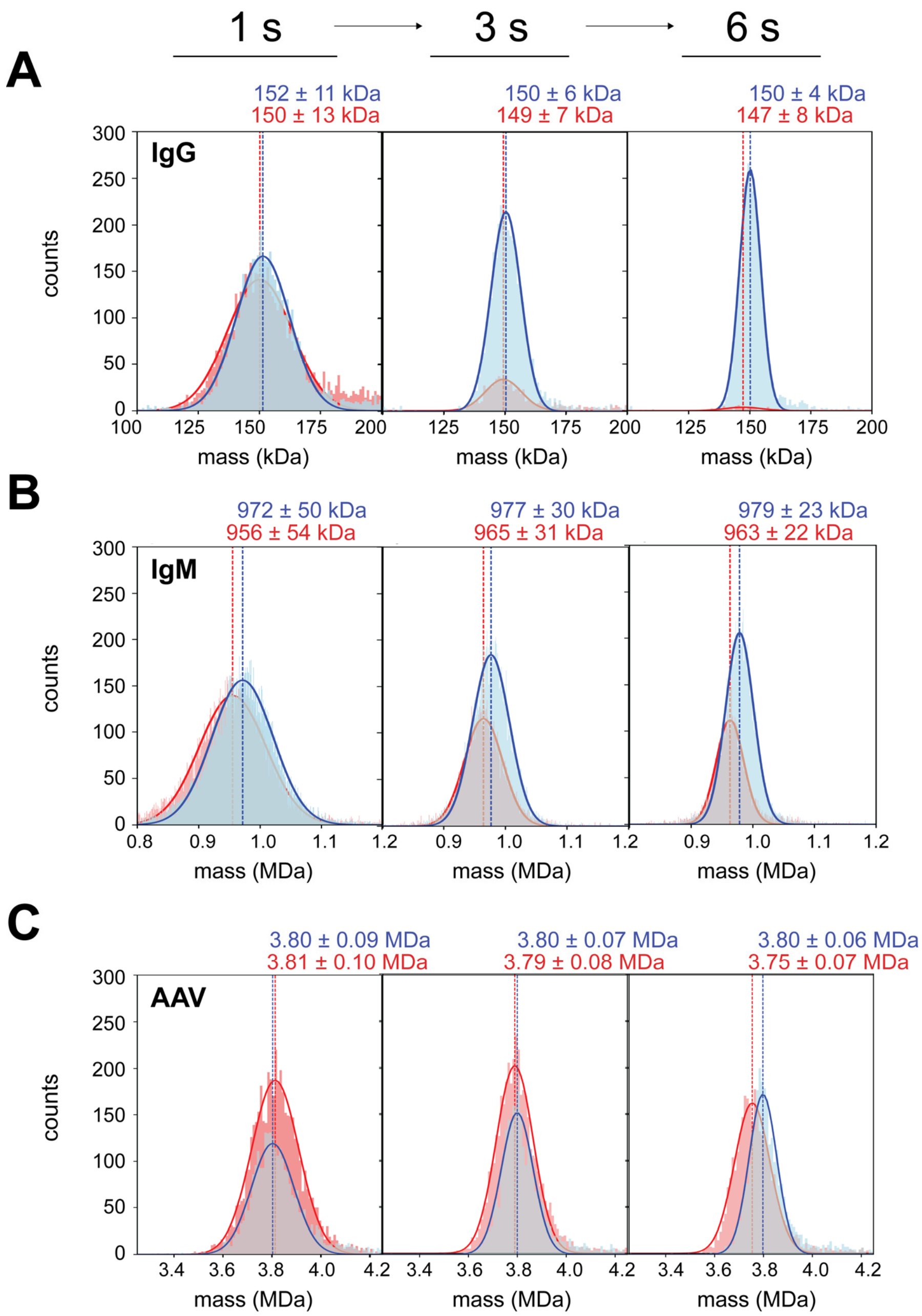

Supplementary Figure 5: Mass histograms at earlier time points in the ultralong transients. Displayed are the mass histograms of **A)** IgG (IgG1-RGY monomer), **B)** IgM and **C)** AAV processed up to 1, 3, and 6 seconds of the ultralong transients. With the latter time points of 3 and 6 seconds, containing at least 4 segments to assess ion stability based on the *m/z* path. For each analyte, the mass histogram is overlaid in red when the valve was switched off and in blue when the valve was switched on. Indicated above are the average mass ± standard deviation of the Gaussian fit. Also, early along the ultralong transient, the benefit of the pulsed valve and its improved vacuum positively affecting ion stability, ion accumulation and mass accuracy.

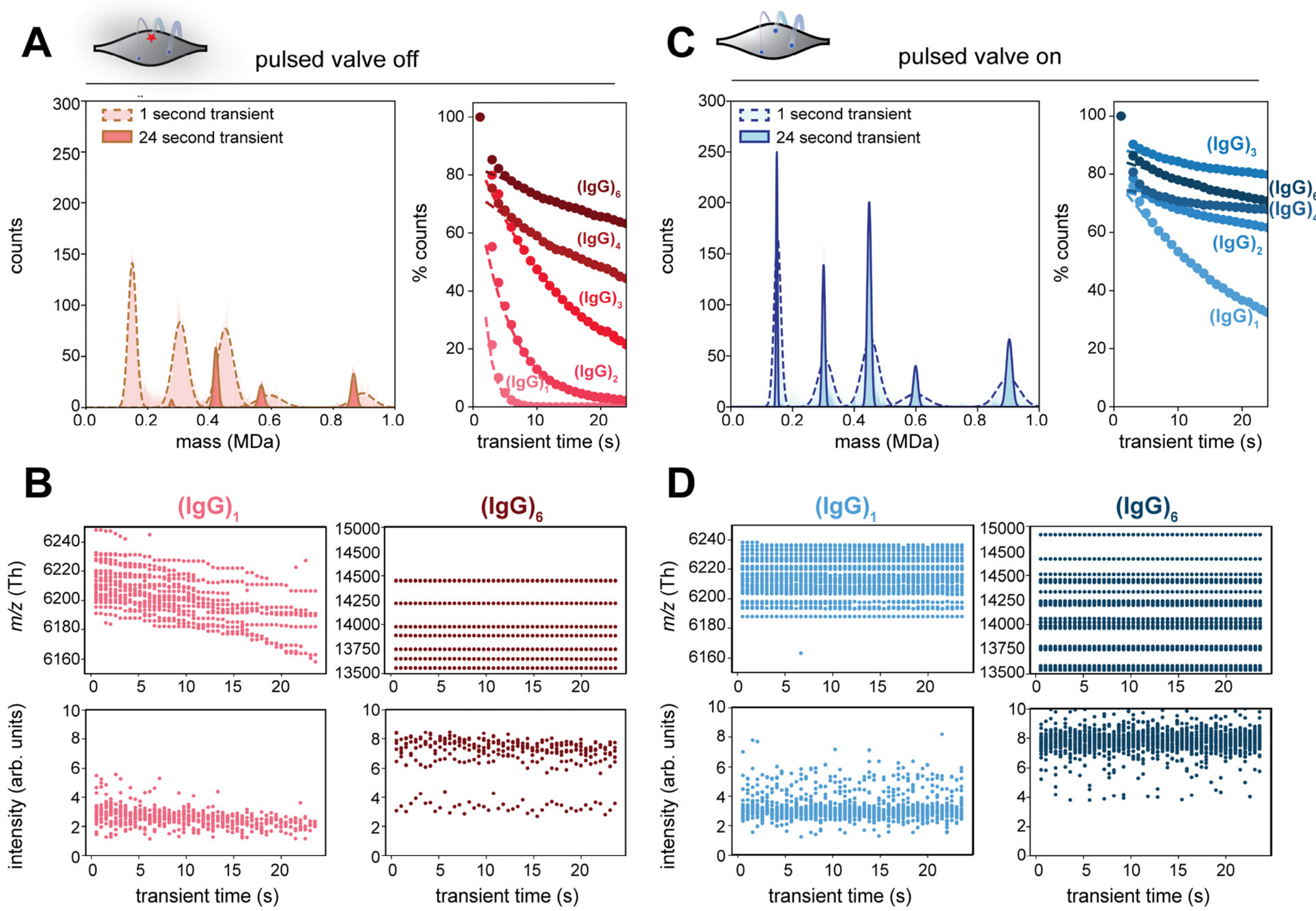

Supplementary Figure 6: Ion stability of IgG1-RGY oligomers without and with the pulsed valve. A) CDMS analysis of IgG1-RGY oligomers with the pulsed valve off (red). Histograms (with Gaussian fits) of the extracted oligomer masses are shown for transients of 1 and 24 seconds, revealing an increase in resolution at 24 seconds but also a substantial loss of $(IgG)_1$ monomers. On the right is depicted the relative number of stable ions over the transient time processed in 1-second increments from 1 to 24 seconds, of which half-life times can be extracted (see Fig. 4). **B)** Close-up of $(IgG)_1$ monomers and $(IgG)_6$ hexamers from an example scan. Ions of the monomer especially drift in both *m/z* (neutral loss) and/or intensity (ion drag), with many ions completely lost during the transient. **C)** By using the pulsed valve (blue), most ions remain stable, and the mass resolution of all IgG species improves along the transient time. **D)** In the zoomed-in view of $(IgG)_1$ and $(IgG)_6$, ions do not substantially drift in *m/z* or intensity. Only occasionally, a loss of an individual ion is observed.

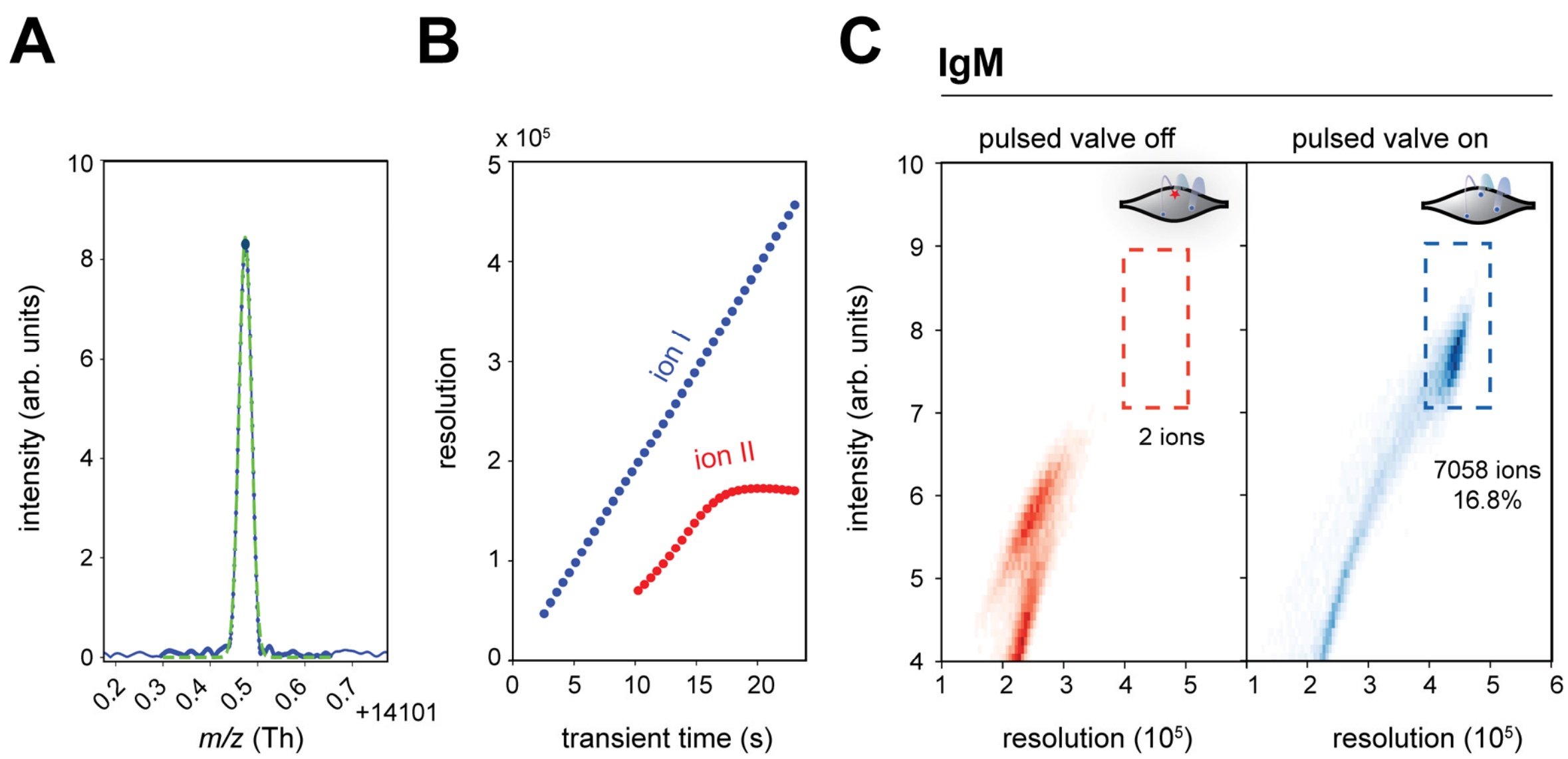

Supplementary Figure 7: The pulsed valve improves obtainable mass resolution in CDMS of individual IgM ions. To assess the quality of IgM CDMS and the pulsed valve without frequency chasing, the improved *m/z* resolution of individual ions was monitored over the transient time as a measure of ion stability. **A)** For each individual ion, the resolution was determined by fitting a Gaussian curve (dashed green line) and taking the peak centroid (blue dot). **B)** Two example ions are shown, indicative of a well-behaved ion with the pulsed valve on (blue) or an unstable ion measured in the absence of the pulsed valve (red). The potential increase in resolution was evaluated by extending the transient analysis every time with a 512-millisecond timestep. Ion I shows a linear increase in resolution over the transient, as expected when not affected by collisions with background gas, while ion II only has a short period of stability with a linear increase in resolution between 10 and 15 seconds of the measurement time. **C)** Taking all ions into account, IgM was measured without (red) or with (blue) the pulsed valve on. In these 2D-histograms, each ion's intensity value is plotted against its *m/z*-resolution. Stable particles that meet conditions for an optimal ion behavior are boxed within the dashed square ($7<\text{intensity}<9$ and $4 \times 10^5<\text{resolution}<5 \times 10^5$). Only with the pulsed valve on does a substantial number of ions meet the intensity and resolution standards expected when extending the transient to 24 seconds.

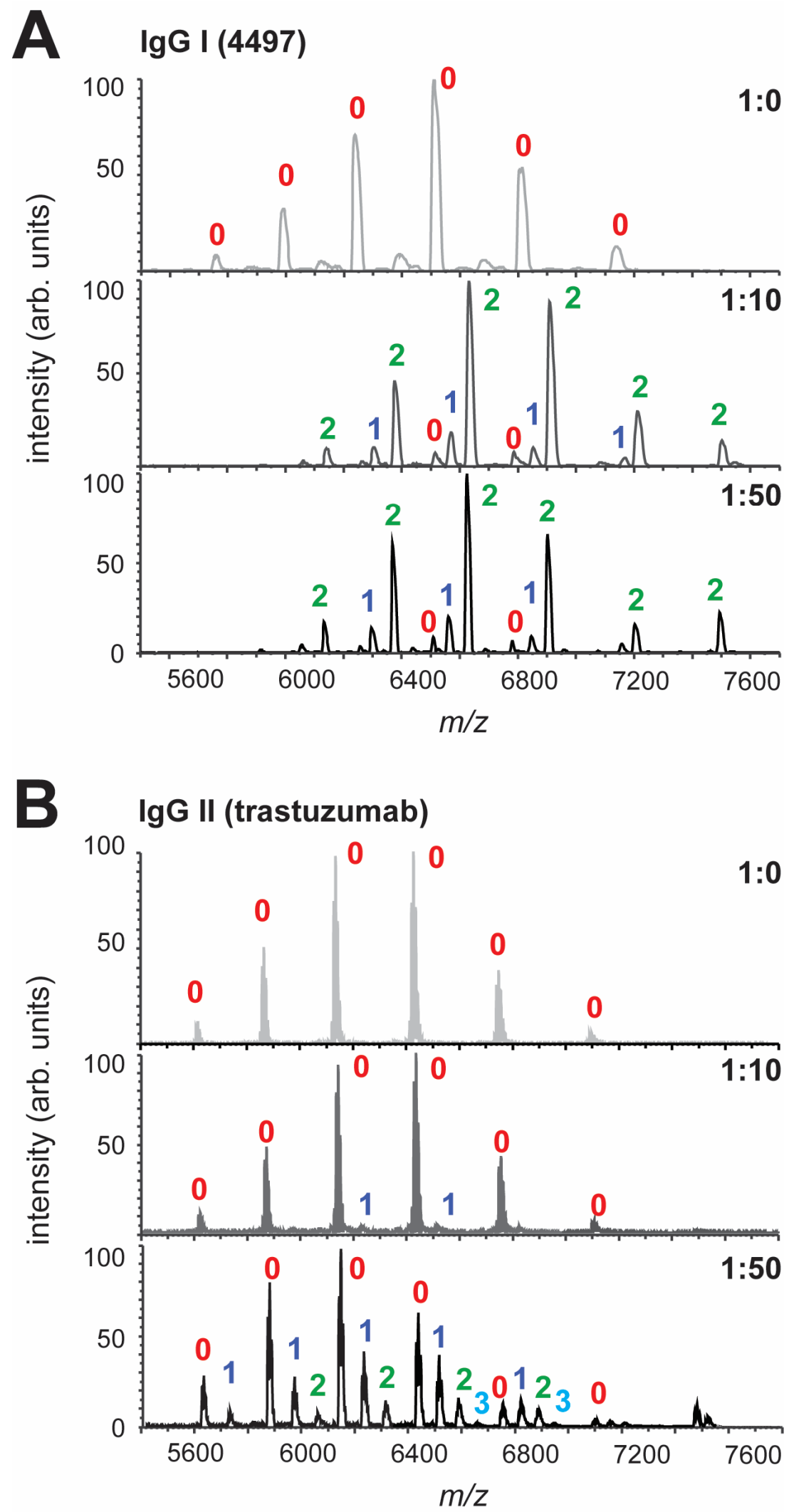

Supplementary Figure 8: Titration experiments of the ~8,5 kDa SpA-B domain peptide with two IgG sequence variants. IgG concentrations were kept constant (IgG I 4497: 2.5 µM; IgG II trastuzumab: 4.4 µM), while SpA-B peptide was added at increasing ratios, starting from 1:0 and progressing up to 1:50. Native ensemble mass spectrometry was used to probe binding of the SpA-B domain to two IgG sequence variants (IgG I 4497 and IgG II trastuzumab). The resulting mass spectra were analyzed to determine the distribution of species containing 0, 1, 2, 3, or 4 bound SpA-B domains. We show the spectra at selected fixed ratios (1:0, 1:10, and 1:50), revealing that at these ratios the IgG I 4497 variant binds with a higher affinity than variant IgG II trastuzumab. **A)** Upon gradual increase of SpA-B concentration, IgG I 4497 binds a maximum of two SpA-B domains. **B)** In contrast, the lower-affinity IgG variant II trastuzumab accommodates up to three SpA-B domains at the highest ratio tested; binding of a fourth domain would likely require higher concentrations, as suggested by CDMS experiments.

Supplementary Table 1: MS and data processing parameters for the measured analytes. The first four columns provide the mass spectrometer desolvation settings, including collision gas type, the pressure setting, in-source trapping, and HCD energy level. The last two columns present the settings used for analyte frequency chasing: segment length and the threshold for the rolling standard deviation to remove ions with unstable frequencies.

	UHMR settings				***Frequency chasing parameters***	
	Collision gas	***Pressure setting***	***In source trapping (V)***	***HCD (V)***	***Segment length (milliseconds)***	***Standard deviation threshold***
rAAVs	xenon	3	- 10	125	256	2
pBR322	nitrogen	2-3	not used	50-60	128	4
IgG-RGY	nitrogen	4	not used	175	512	0.2
IgM	nitrogen	4.5	not used	125-150	512	0.5
IgG+SpA	nitrogen	2-2.5	not used	80-90	512	1.5
