# Supplementary material for: Modulating pressure in the Orbitrap improves sensitivity and mass resolution in charge detection mass spectrometry
Source: Nat Commun. 2026 Aug 13;17:9690. doi: 10.1038/s41467-026-76691-1 (PMC13558686; doi:10.1038/s41467-026-76691-1)
Supplement: Supplementary file 2 [file 41467_2026_76691_MOESM2_ESM.pdf]

nature portfolio

Corresponding author(s): Albert J. R. Heck

Last updated by author(s): 22/06/26

Reporting Summary

Nature Portfolio wishes to improve the reproducibility of the work that we publish. This form provides structure for consistency and transparency in reporting. For further information on Nature Portfolio policies, see our Editorial Policies and the Editorial Policy Checklist.

Statistics

For all statistical analyses, confirm that the following items are present in the figure legend, table legend, main text, or Methods section.

n/a	Confirmed	
☐	☒	The exact sample size (n) for each experimental group/condition, given as a discrete number and unit of measurement
☐	☒	A statement on whether measurements were taken from distinct samples or whether the same sample was measured repeatedly
☒	☐	The statistical test(s) used AND whether they are one- or two-sided<br>*Only common tests should be described solely by name; describe more complex techniques in the Methods section.*
☒	☐	A description of all covariates tested
☒	☐	A description of any assumptions or corrections, such as tests of normality and adjustment for multiple comparisons
☐	☒	A full description of the statistical parameters including central tendency (e.g. means) or other basic estimates (e.g. regression coefficient) AND variation (e.g. standard deviation) or associated estimates of uncertainty (e.g. confidence intervals)
☒	☐	For null hypothesis testing, the test statistic (e.g. F, t, r) with confidence intervals, effect sizes, degrees of freedom and P value noted<br>*Give P values as exact values whenever suitable.*
☒	☐	For Bayesian analysis, information on the choice of priors and Markov chain Monte Carlo settings
☒	☐	For hierarchical and complex designs, identification of the appropriate level for tests and full reporting of outcomes
☒	☐	Estimates of effect sizes (e.g. Cohen's d, Pearson's r), indicating how they were calculated

Our web collection on statistics for biologists contains articles on many of the points above.

Software and code

Policy information about availability of computer code

Data collection	Custom MS tune Orbitrap Q Exactive™ UHMR, FTMS Booster X2 2023.1.0
Data analysis	Peak-by-Peak version 2024.05.0, in-house developed Jupyter Notebook with associated Python 3

For manuscripts utilizing custom algorithms or software that are central to the research but not yet described in published literature, software must be made available to editors and reviewers. We strongly encourage code deposition in a community repository (e.g. GitHub). See the Nature Portfolio guidelines for submitting code & software for further information.

Data

Policy information about availability of data

All manuscripts must include a data availability statement. This statement should provide the following information, where applicable:

- Accession codes, unique identifiers, or web links for publicly available datasets
- A description of any restrictions on data availability
- For clinical datasets or third party data, please ensure that the statement adheres to our policy

Data supporting the findings of this study are available within the paper and its Supplementary Information. Source data are provided with this paper. Alternatively, the data is available from the corresponding author upon request. An example dataset is provided alongside the example code. An example Python script for long transient data processing is available in the Zenodo database under DOI 10.5281/zenodo.20690164.

Research involving human participants, their data, or biological material

Policy information about studies with human participants or human data. See also policy information about sex, gender (identity/presentation), and sexual orientation and race, ethnicity and racism.

Reporting on sex and gender	n/a no human research participants were used in this work.
Reporting on race, ethnicity, or other socially relevant groupings	n/a no human research participants were used in this work.
Population characteristics	n/a no human research participants were used in this work.
Recruitment	n/a no human research participants were used in this work.
Ethics oversight	n/a no human research participants were used in this work.

Note that full information on the approval of the study protocol must also be provided in the manuscript.

Field-specific reporting

Please select the one below that is the best fit for your research. If you are not sure, read the appropriate sections before making your selection.

[x] Life sciences [ ] Behavioural & social sciences [ ] Ecological, evolutionary & environmental sciences

For a reference copy of the document with all sections, see nature.com/documents/nr-reporting-summary-flat.pdf

Life sciences study design

All studies must disclose on these points even when the disclosure is negative.

Sample size	Several hundred to thousands of ions were measured to ensure adequate signal-to-noise.
Data exclusions	Ions with unstable trajectories were filtered as described in the material and methods section with different strategies depending on the data processing (m/z trajectory, intensity and resolution filtering), as detailed in the Methods section, and ion counts are reported throughout the Figures and Results section.
Replication	Replicates represents unique, independent measurements, as detailed in the Methods section, and ion counts are reported throughout the Figures and Results section.
Randomization	n/a no randomization was applicable since no organization in experimental groups.
Blinding	n/a no blinding was applicable since no organization in experimental gorups.

Reporting for specific materials, systems and methods

We require information from authors about some types of materials, experimental systems and methods used in many studies. Here, indicate whether each material, system or method listed is relevant to your study. If you are not sure if a list item applies to your research, read the appropriate section before selecting a response.

Materials & experimental systems

n/a	Involved in the study	
[ ]	[x]	Antibodies
[x]	[ ]	Eukaryotic cell lines
[x]	[ ]	Palaeontology and archaeology
[x]	[ ]	Animals and other organisms
[x]	[ ]	Clinical data
[x]	[ ]	Dual use research of concern
[x]	[ ]	Plants

Methods

n/a	Involved in the study	
[x]	[ ]	ChIP-seq
[x]	[ ]	Flow cytometry
[x]	[ ]	MRI-based neuroimaging

Antibodies

Antibodies used	Trastuzumab (Roche), IgG-4497 and Strep IgM (University Medical Center Utrecht, Utrecht, Netherlands), IgG1-RGY Genmab (Utrecht Netherlands),
Validation	n/a

Plants

Seed stocks	n/a
Novel plant genotypes	n/a
Authentication	n/a
