# Supplementary material for: Modulating pressure in the Orbitrap improves sensitivity and mass resolution in charge detection mass spectrometry
Source: Nat Commun. 2026 Aug 13;17:9690. doi: 10.1038/s41467-026-76691-1 (PMC13558686; doi:10.1038/s41467-026-76691-1)
Supplement: Supplementary file 3 [file 41467_2026_76691_MOESM3_ESM.pdf]

nature portfolio

Peer Review File

Modulating pressure in the Orbitrap improves sensitivity and mass resolution in charge detection mass spectrometry

Corresponding Author: Professor Albert Heck

This file contains all reviewer reports in order by version, followed by all author rebuttals in order by version.

Version 0:

Reviewer comments:

Reviewer #1

(Remarks to the Author)
In this manuscript the authors present a solution to a previously foundational limitation of the Orbitrap UHMR system - the leaking of HCD cell gas into the orbitrap/UHV region. By incorporating a pulse of gas, timed with the injection of ions into the HCD cell, the authors are able to extract the benefits of high-pressure trapping (collisional focusing, adduct removal) while maintaining low orbitrap pressures optimal for CDMS analysis. The low orbitrap pressures enable very long CDMS acquisitions without the typical collisions experienced when operating at higher pressures. This pressure-alternating regime enables 1) more viable, stable, large ions to be trapped and observed across 24s transients and 2) enhanced survival of small (~150kDa) ions in the orbitrap.

Overall the authors present a step increase in the performance of the UHMR Orbitrap platform for CDMS analysis, mitigating a key issue that limited its performance. While I have concerns about the reproducibility of the gas control, the ways the data are compared, and some under-discussed limitations, the improvements made to this system are impactful and will be of great benefit to the CDMS community. Once my comments are addressed, I believe this manuscript will be a strong addition to Nature Communications.

1. The authors consistently compare data produced by 24s transients with the gas pulse on vs. off. While this is certainly necessary to show the benefits of the instrument modification and characterize ion stability changes over the transient, it seems much more relevant to compare the 24s transients from the updated instrument with the optimal transient length available on the standard setup for ions which produce essentially noise at 24s transients. This is especially relevant for the IgG1 and plasmid DNA samples:
a. On p21 line 324 the authors state that because the IgG1 ions do not survive 24s of Orbitrap trapping it is “impossible to determine their mass by CDMS.” This statement obfuscates the clear mass determination one can get for an IgG1 at shorter transients, as shown in figure S4A. A much more useful comparison would be the best performance for an IgG1 with the pulsed valve off (S4A, 1s transient) vs. pulsed valve on at the same transient, and then at the much longer transients enabled by the low UHV pressure. Figure S5b nicely shows this type of comparison (for an IgM): at equivalent transients there is a resolution boost with the pulsed valve (which there seems to be on the IgG sample as well), and this resolution can be extended with longer transients due to the improved pressure control.
b. For the plasmid DNA vignette the reader is left guessing what the resolution improvement would be for this sample if it were attempted on the standard instrument with the short transients necessary to obtain usable CDMS results. Using the <128 ms transients the authors state are necessary for analysis of this sample class on the standard instrument (p11 line 180), can one acquire usable data?

2. Regarding the solenoid valve performance and Fig 1/S1B:
a. Can the authors comment on the reproducibility of the gas pressure during the “puff” phase? Visually it is surprisingly variable in Fig S1B, though this may be due to the poor temporal resolution of this measurement.
b. Is there an upper limit on the pressure one is able to spike into the HCD cell, or can one generate temporary pressures that mirror the range set by the pressure setting in the standard instrument setup?
c. Is the pressure in the UHV region stabilized prior to the start of the 25s CDMS scan, and are any undesired collisions measurable if not?

3. The time impact of the implementation of the pulsed valve on p21 lines 320-323 seems under-discussed. My two main

concerns are:
a. For ions which were already viable with the pulsed valve off, does the pulsed valve enable faster acquisition to obtain the same resolution/ion counts/data quality?
b. How long did each of these experiments take? I can not find any quantitative statements on the overall time cost of these measurements.

4. Some discussion of the unique data processing used for the pBR322 sample would be beneficial. For ions which do not stabilize until 3-10 seconds in the orbitrap – do the authors suspect this is due to insufficient desolvation prior to orbitrap analysis or some other phenomenon?

5. The different intensity scaling for the 2 instrument setups in Fig 2C makes it seem like the pulsed valve off data are much lower intensity than the pulsed valve on data. Matching the axes of the 2 datasets being compared would remove this confusion.

Reviewer #2

(Remarks to the Author)
Heck and co-workers, one of the leading labs in Orbitrap-based charge detection mass spectrometry (CDMS), present an elegant yet relatively straightforward instrumental modification that significantly enhances the fidelity of single-ion analysis in Orbitrap detectors. The work addresses a fundamental conundrum in native CDMS: efficient transmission and stabilization of large macromolecular ions typically require relatively high pressures within the mass spectrometer to promote collisional cooling, yet leakage of this high-pressure gas into the Orbitrap analyzer destabilizes ion trajectories and compromises the long transient recordings required for precise single-ion detection. The authors introduce an intuitive pressure-control strategy that mitigates this limitation, thereby improving ion stability during detection and expanding the practical scope of Orbitrap-based CDMS. The presented data convincingly demonstrate the impact of this approach, particularly for the analysis of large biomolecular assemblies such as virus-like particles and IgG complexes. Moreover, experiments with nucleic acid–containing complexes illustrate how reduced Orbitrap pressure conditions help preserve labile assemblies that would otherwise dissociate under conventional conditions during the long transient time. Together, these results represent an important technological advance for native CDMS measurements of heterogeneous macromolecular complexes.

Nevertheless, several questions arise that could further strengthen the manuscript.

First, the authors emphasize the benefits of their approach primarily for ultra-long transient acquisitions (up to 25 s). While impressive, many biomolecular systems—particularly transient protein–protein or protein–ligand interactions—may not remain stable over such long detection windows. Importantly, even in more typical commercial Orbitrap CDMS experiments using ~2 s transients, pressure-induced trajectory instability can lead to ion loss, incorrect charge assignment, and uncertainty in resolving ligand-bound satellite peaks on large complexes. Demonstrating that the proposed pulsed-gas low-pressure strategy also improves charge accuracy and reduces mass assignment errors under these shorter transient conditions would substantially broaden the impact of the work.

Second, improved ion survival at reduced Orbitrap pressures may enhance the sensitivity of image-current detection. This could enable detection of lower charge states—currently difficult to detect below ~6+ in UHMR-based CDMS—and might also reduce the acquisition time required to obtain statistically meaningful datasets. If experimentally demonstrated, these advantages would further extend the analytical reach of Orbitrap CDMS.

Third, the current implementation relies on a UHMR instrument coupled to the FTMS Booster to enable ultra-long transient acquisition. It would therefore be valuable to assess how broadly applicable this pressure-modulation approach is to Orbitrap CDMS platforms that lack such long-transient capabilities.

Finally, the pulsed valve configuration's duty-cycle implications warrant further discussion. Specifically, a quantitative analysis of how Orbitrap vacuum recovery depends on buffer time, including the minimum injection-time/buffer-time ratio required for stable operation, would help define the practical lower limits for transient duration at which this strategy remains beneficial.

A few minor points should also be addressed:

The authors report that the gas pulse valve implementation enabled a corrected rAAV mass measurement of 3.80 MDa compared with a previously reported 3.62 MDa; it would be useful to cite references that establish the expected theoretical mass for comparison.
In addition, clarification would be helpful regarding why the IgG-to-SPA binding ratios differ among the analyzed variants.

It would also be useful to elaborate further on the integration of the controller chip with the HCD setup and how the triggering mechanism is controlled. This would help other users implement similar strategies in their instruments, broadening the use of the proposed approach.

Overall, despite these questions, the work represents a significant step forward in improving the robustness and analytical performance of Orbitrap-based native CDMS.

Reviewer #3

(Remarks to the Author)
In this manuscript, Ebberink et al. describe a hardware modification to a QExactive UHMR Orbitrap mass spectrometer to enable pulsed pressure modulation between the gas supply and the HCD cell. This modification resolves the pressure mismatch in Orbitrap-based CDMS by allowing high-pressure desolvation/activation in the HCD while subsequently decreasing the pressure in the Orbitrap during ultralong acquisitions. This mismatch has been a hindrance in Orbitrap-based CDMS as it reduces the stability and therefore accuracy of m/z and z measurements and so this modification represents a necessary step forward in advancing this technology. The authors describe several examples where pulsed pressure modulation improves accuracy, including AAVs and antibodies, which are of broad interest to most users of this technology. Overall, the implementation is well executed, the data are clearly presented, and the improvements are significant. However, I have minor comments to be addressed:

-In the introduction, page 4 line 83 reads “For such low m/z, high frequency ions, the likelihood of collisions with residual gas molecules is high, and due to their low mass, the impact of such collisions is elevated.” While the latter point is valid, the first part may require clarification. Since mean free path is inversely proportional to particle size, a low m/z (presumably smaller) ion would not necessarily be more likely to undergo collisions, although the effects of such collisions may indeed be more significant. Please clarify or revise this statement.

-In Figure 4B, the improvement in ion half-life appears to diminish beyond a certain oligomer size. Could the authors comment on the mechanistic basis for this trend? In particular, it would be helpful to provide a more generalizable discussion relating ion size and mean free path at this pressure regime, and how these factors translate into improvements and/or scale with molecular weight.

-Page 14, line 222, similarly notes that the improvement in ion lifetime becomes less pronounced as molecular weight increases. This trend warrants further explanation. Are there specific classes of analytes for which this approach meaningfully extends accessibility, particularly in cases where collisions with background gas would otherwise be prohibitively detrimental?

-In Figure 5, the caption appears to be missing a word in the phrase “residues in red divergence from the suspected binding sequence.” Please revise for clarity. In addition, what do the annotations in the figure “(1:73)” and “(1:94)” refer to? Do these represent molar ratios? Please clarify in the figure caption or main text.

-Further to this, according to the methods section, the peptide concentration used (70 µM) is quite high and could potentially promote nonspecific binding or artifacts. The authors should clarify how nonspecific interactions were ruled out. This could be addressed either by providing a quantitative justification (e.g., probability calculations) or by including a peptide titration experiment to demonstrate that the additional binding observed is not nonspecific.

Overall, the manuscript presents a clever hardware modification supported by compelling examples. However, its impact for a broader readership would be strengthened by a clearer articulation of the generalizable advantages of the approach (e.g., the molecular weight range over which it is most beneficial and why that is so).

Version 1:

Reviewer comments:

Reviewer #1

(Remarks to the Author)
The authors have made edits to the main text to better characterize the advantages and limitations imposed by this hardware modification. They have also added supplemental figures S5 and S3 to address my most pressing comments in full. While it is unfortunate that they are unable to obtain a higher fidelity readout of the gas pressure, they have addressed this in the text. As such, I have no further comments and recommend publication.

Reviewer #2

(Remarks to the Author)
The Authors have satisfactorily addressed all my questions

Reviewer #3

(Remarks to the Author)
The authors have sufficiently addressed my concerns and have improved the manuscript. I have no further queries and find the manuscript suitable for publication.

Open Access This Peer Review File is licensed under a Creative Commons Attribution 4.0 International License, which permits use, sharing, adaptation, distribution and reproduction in any medium or format, as long as you give appropriate credit to the original author(s) and the source, provide a link to the Creative Commons license, and indicate if changes were made.
In cases where reviewers are anonymous, credit should be given to 'Anonymous Referee' and the source.
The images or other third party material in this Peer Review File are included in the article's Creative Commons license, unless indicated otherwise in a credit line to the material. If material is not included in the article's Creative Commons license and your intended use is not permitted by statutory regulation or exceeds the permitted use, you will need to obtain permission directly from the copyright holder.
To view a copy of this license, visit https://creativecommons.org/licenses/by/4.0/

Point-by-point response to the reviewers' comments

Reviewer comments in black, our responses in blue

Reviewer #1 (Remarks to the Author):

In this manuscript the authors present a solution to a previously foundational limitation of the Orbitrap UHMR system - the leaking of HCD cell gas into the orbitrap/UHV region. By incorporating a pulse of gas, timed with the injection of ions into the HCD cell, the authors are able to extract the benefits of high-pressure trapping (collisional focusing, adduct removal) while maintaining low orbitrap pressures optimal for CDMS analysis. The low orbitrap pressures enable very long CDMS acquisitions without the typical collisions experienced when operating at higher pressures. This pressure-alternating regime enables 1) more viable, stable, large ions to be trapped and observed across 24s transients and 2) enhanced survival of small (~150kDa) ions in the orbitrap.

Overall the authors present a step increase in the performance of the UHMR Orbitrap platform for CDMS analysis, mitigating a key issue that limited its performance. While I have concerns about the reproducibility of the gas control, the ways the data are compared, and some under-discussed limitations, the improvements made to this system are impactful and will be of great benefit to the CDMS community. Once my comments are addressed, I believe this manuscript will be a strong addition to Nature Communications.

We thank the reviewer for this strong endorsement of our work.

1. The authors consistently compare data produced by 24s transients with the gas pulse on vs. off. While this is certainly necessary to show the benefits of the instrument modification and characterize ion stability changes over the transient, it seems much more relevant to compare the 24s transients from the updated instrument with the optimal transient length available on the standard setup for ions which produce essentially noise at 24s transients. This is especially relevant for the IgG1 and plasmid DNA samples:
a. On p21 line 324 the authors state that because the IgG1 ions do not survive 24s of Orbitrap trapping it is "impossible to determine their mass by CDMS." This statement obfuscates the clear mass determination one can get for an IgG1 at shorter transients, as shown in figure S4A. A much more useful comparison would be the best performance for an IgG1 with the pulsed valve off (S4A, 1s transient) vs. pulsed valve on at the same transient, and then at the much longer transients enabled by the low UHV pressure. Figure S5b nicely shows this type of comparison (for an IgM): at equivalent transients there is a resolution boost with the pulsed valve (which there seems to be on

the IgG sample as well), and this resolution can be extended with longer transients due to the improved pressure control.

The reviewer raises an interesting point by focusing on shorter transients rather than considering the entire 24s transient. However, the ability to process short transients (< 1s) on molecules such as IgG is limited. Because native IgG ions carry fewer charges, achieving a good signal-to-noise ratio requires a transient time of at least 512 ms. Still, addressing the reviewers point, we added in the revised manuscript now also data for recordings at 1, 3, and 6 seconds adding a new supplementary figure (Fig. S5). The data in this figure demonstrate that also at these shorter transient times (with 1 and 3 sec achievable on standard UHMR instruments), switching on the valve already benefits ion stability and mass accuracy. We refer towards this new supplementary figure 5 in the revised manuscript on page 14, line 219.

b. For the plasmid DNA vignette the reader is left guessing what the resolution improvement would be for this sample if it were attempted on the standard instrument with the short transients necessary to obtain usable CDMS results. Using the <128 ms transients the authors state are necessary for analysis of this sample class on the standard instrument (p11 line 180), can one acquire usable data?

In response to the reviewer we added a new supplementary figure showing a CDMS measurement of pBR322, using an unmodified mass spectrometry setup with a short transient time (64 ms). This measurement indeed indicates that data on DNA can be acquired with conventional CDMS, although it comes as the expense of exhibiting broad peaks and high background interference due to DNA fragmentation (shearing during the ESI process). We refer in the revised manuscript to this new supplementary figure 3 on page 11 line 183.

2. Regarding the solenoid valve performance and Fig 1/S1B:
a. Can the authors comment on the reproducibility of the gas pressure during the "puff" phase? Visually it is surprisingly variable in Fig S1B, though this may be due to the poor temporal resolution of this measurement.
b. Is there an upper limit on the pressure one is able to spike into the HCD cell, or can one generate temporary pressures that mirror the range set by the pressure setting in the standard instrument setup?

As the reviewer points out, there is indeed a poor resolution in the measurement of the pressure. The pressure gauge only allows for a read-out every few seconds. Therefore, we are unable to determine the exact height of the pressure peak during the short 'puff'. Because the valve only opens and closes, and does not influence the pressure setting, the pressure in the HCD cell is presumably limited by the pressure setting, as in a

standard instrument setup. We expect that the achieved pressures mirror indeed the range set by the pressure setting in the standard setup, but can unfortunately not directly measure that.

c. Is the pressure in the UHV region stabilized prior to the start of the 25s CDMS scan, and are any undesired collisions measurable if not?

It is possible to stabilize the pressure before starting the scan, then switch the pulsed valve on to achieve a vacuum during the long transient. However, this requires the scan before the intended 'low vacuum' scan to have the *pulsed valve off* for the entirety of that scan. In effect, the scans then alternate between high and low pressure. This can be interesting for studying the effect of the valve within a single measurement. However, it takes twice as long to then obtain an optimized low-pressure, long-transient measurement, since only half the scans can be used.

In fact, such pressure stabilization by alternating between scans with the valve on and off was used for our DNA measurements. Despite the stabilization of the pressure, we noticed an effect of the somewhat higher pressure at the start of the scan, including some undesirable collisions leading to loss of ions. Apparently, the pump-down time from high to low pressure and the desolvation of the analyte (more difficult for DNA) have a greater influence than pressure stabilization. To better point towards this issue, we added the sentence '*In case of instability, it occurs mostly at the start of the scan when the optimal vacuum conditions have not yet been reached*' on page 11, lines 191-193.

3. The time impact of the implementation of the pulsed valve on p21 lines 320-323 seems under-discussed. My two main concerns are:
a. For ions which were already viable with the pulsed valve off, does the pulsed valve enable faster acquisition to obtain the same resolution/ion counts/data quality?

For ions that can readily be measured with conventional CDMS, the improvement in ion counts and mass accuracy is evident early on, when the pulsed valve is switched on. For instance, with AAVs commonly measured with CDMS, the average mass begins to shift and become inaccurate within 3 to 6 seconds due to background collisions under typical conditions. With the new pulsed valve switched on, this artifactual shift does not occur. This can be observed more clearly in the new supplementary Figure 5 that is included in the revision.

b. How long did each of these experiments take? I can not find any quantitative statements on the overall time cost of these measurements.

Typically, full measurements ranged from between 45 to 90 minutes. We modified the sentence on page 23 line 383 to mention this.

4. Some discussion of the unique data processing used for the pBR322 sample would be beneficial. For ions which do not stabilize until 3-10 seconds in the orbitrap – do the authors suspect this is due to insufficient desolvation prior to orbitrap analysis or some other phenomenon?

Indeed, as the reviewer points out, it is likely that DNA ions remain quite solvated following the ESI process and retain also salts in line with what is described in earlier studies (ref 26 and 46). Therefore, with some (residual) elevated pressure at the start of the scan, the DNA ions can remain somewhat unstable within the first seconds. We now commented on this point in the revised manuscript on page 11, lines 191-193.

5. The different intensity scaling for the 2 instrument setups in Fig 2C makes it seem like the pulsed valve off data are much lower intensity than the pulsed valve on data. Matching the axes of the 2 datasets being compared would remove this confusion.

We thank the reviewer for pointing this out and modified the axis in Fig 2C in the revised manuscript.

Reviewer #2 (Remarks to the Author):

Heck and co-workers, one of the leading labs in Orbitrap-based charge detection mass spectrometry (CDMS), present an elegant yet relatively straightforward instrumental modification that significantly enhances the fidelity of single-ion analysis in Orbitrap detectors. The work addresses a fundamental conundrum in native CDMS: efficient transmission and stabilization of large macromolecular ions typically require relatively high pressures within the mass spectrometer to promote collisional cooling, yet leakage of this high-pressure gas into the Orbitrap analyzer destabilizes ion trajectories and compromises the long transient recordings required for precise single-ion detection. The authors introduce an intuitive pressure-control strategy that mitigates this limitation, thereby improving ion stability during detection and expanding the practical scope of Orbitrap-based CDMS. The presented data convincingly demonstrate the impact of this approach, particularly for the analysis of large biomolecular assemblies such as virus-like particles and IgG complexes. Moreover, experiments with nucleic acid–containing complexes illustrate how reduced Orbitrap pressure conditions help preserve labile assemblies that would otherwise dissociate under conventional conditions during the long transient time. Together, these results represent an important technological advance for native CDMS measurements of heterogeneous macromolecular complexes.

We thank the reviewer for this strong endorsement of our work.

Nevertheless, several questions arise that could further strengthen the manuscript.

First, the authors emphasize the benefits of their approach primarily for ultra-long transient acquisitions (up to 25 s). While impressive, many biomolecular systems—particularly transient protein–protein or protein–ligand interactions—may not remain stable over such long detection windows. Importantly, even in more typical commercial Orbitrap CDMS experiments using ~2 s transients, pressure-induced trajectory instability can lead to ion loss, incorrect charge assignment, and uncertainty in resolving ligand-bound satellite peaks on large complexes. Demonstrating that the proposed pulsed-gas low-pressure strategy also improves charge accuracy and reduces mass assignment errors under these shorter transient conditions would substantially broaden the impact of the work.

The reviewers point is similar as the point raised by reviewer 1, therefore, our response is also similar. In the revised manuscript we have aimed to display more insight into the benefits of the pulsed valve at shorter transient times. Therefore, we added a new supplementary figure 5 focusing subsequently on transients of 1, 3, and 6 seconds analyzing subsequently IgG1, IgM, and AAV. Looking at the mass histograms from these early time points (3 and 6 seconds) demonstrates that switching the pulsed valve on has considerable benefits for ion stability, resulting in a more stable mass and better mass accuracy. Especially for the smallest IgG1 particles, the ion loss due to unwanted background collisions with the switch off becomes apparent.

Second, improved ion survival at reduced Orbitrap pressures may enhance the sensitivity of image-current detection. This could enable detection of lower charge states—currently difficult to detect below ~6+ in UHMR-based CDMS—and might also reduce the acquisition time required to obtain statistically meaningful datasets. If experimentally demonstrated, these advantages would further extend the analytical reach of Orbitrap CDMS.

The reviewer makes an excellent point that using the pulsed valve for very low-charged ions could also be very helpful. However, we feel that the detection of such low-charge or singly-charged ions ($z < 6$) for CDMS is a project in its own right that we indeed aim to work on in the future. Detection of only a few charges or a single charge requires a different data-processing approach, as they produce signals that are so low that they are difficult to distinguish from the noise level. Therefore, we feel that it is out of scope of the current manuscript.

Third, the current implementation relies on a UHMR instrument coupled to the FTMS Booster to enable ultra-long transient acquisition. It would therefore be valuable to assess how broadly applicable this pressure-modulation approach is to Orbitrap CDMS platforms that lack such long-transient capabilities.

Especially for smaller, more fragile ions (IgG) or difficult-to-desolvate ions (IgM), better vacuum control is advantageous for ion stability also at shorter transients, up to 3 seconds. Therefore, also without the FTMS Booster, the valve can be used to improve measurements. We point to the potential use of the valve in the absence of an FTMS Booster on page 21, lines 338-342, and to the new supplementary figure 5, which focuses on the earlier timepoints in the transient.

Finally, the pulsed valve configuration's duty-cycle implications warrant further discussion. Specifically, a quantitative analysis of how Orbitrap vacuum recovery depends on buffer time, including the minimum injection-time/buffer-time ratio required for stable operation, would help define the practical lower limits for transient duration at which this strategy remains beneficial.

In our set-up, the pressure can only be measured every few seconds. However, we can observe that at the start, a strong drop in pressure is reached, followed by a slower phase, pumping down the residual gas. The effect of the earlier, larger drop in pressure is immediately evident in the m/z traces of the ions (Fig. S4, S6 and in Fig. 1). For instance, in the first few segments of IgG1, improved ion stability can be observed (Fig. S6B and S6D). Also, in the new supplementary figure 5, which focuses on the early time points of the long transient, we observe improved mass analysis at around 3 to 4 seconds. Therefore, the lower limit would currently be within the same time range (~3 seconds). We now point towards this in the caption of Supplementary Figure 1 and in the discussion on page 21, lines 338-342.

A few minor points should also be addressed:

The authors report that the gas pulse valve implementation enabled a corrected rAAV mass measurement of 3.80 MDa compared with a previously reported 3.62 MDa; it would be useful to cite references that establish the expected theoretical mass for comparison.

The mass of 3.62 MDa stems from the measurement taken with the pulsed valve switched off. Due to background collisions, the intensity value of the ions starts to gradually shift, causing an artificial shift in intensity and thus number of charges and the final mass. Therefore, a mass assignment of 3.62 MDa is incorrect compared to the 3.8 MDa when this intensity drift does not occur. Unfortunately, comparing these numbers to a theoretical mass of an rAAV preparation is difficult because it depends on the stoichiometry of the three capsid proteins. This ratio can vary per serotype and even per lot of rAAV preparations. In our previous work (ref 41), we examined the issue of

intensity drift in depth and measured the same rAAV serotype with a ~3.8 MDa mass assignment. We added this reference data to our revised manuscript.

In addition, clarification would be helpful regarding why the IgG-to-SPA binding ratios differ among the analyzed variants.

As explained in the original manuscript this is primarily due to difference in the sequence of both used IgG constructs as depicted in Figure 5A. These differences in the sequences affect binding stoichiometry and overall binding affinity. The data in Figure 5 are taken in high access of SpA. We have now added a SpA titration experiment in the revised manuscript. The result of the SpA titration is shown in the new Supplementary Figure 8.

It would also be useful to elaborate further on the integration of the controller chip with the HCD setup and how the triggering mechanism is controlled. This would help other users implement similar strategies in their instruments, broadening the use of the proposed approach.

The trigger occurs when ions are purged from the HCD cell, which is accompanied by a steep voltage drop in the HCD cell. This voltage drop is used as a trigger for the Arduino controller chip.

Overall, despite these questions, the work represents a significant step forward in improving the robustness and analytical performance of Orbitrap-based native CDMS.

Again, we thank the reviewer for the strong support and expert raised issues that has helped us to make the presentation clearer and thus our manuscript better

Reviewer #3 (Remarks to the Author):

In this manuscript, Ebberink et al. describe a hardware modification to a QExactive UHMR Orbitrap mass spectrometer to enable pulsed pressure modulation between the gas supply and the HCD cell. This modification resolves the pressure mismatch in Orbitrap-based CDMS by allowing high-pressure desolvation/activation in the HCD while subsequently decreasing the pressure in the Orbitrap during ultralong acquisitions. This mismatch has been a hindrance in Orbitrap-based CDMS as it reduces the stability and therefore accuracy of m/z and z measurements and so this modification represents a necessary step forward in advancing this technology. The authors describe several examples where pulsed pressure modulation improves accuracy, including AAVs and antibodies, which are of broad interest to most users of

this technology. Overall, the implementation is well executed, the data are clearly presented, and the improvements are significant. However, I have minor comments to be addressed:

We thank the reviewer for the strong support.

-In the introduction, page 4 line 83 reads “For such low m/z, high frequency ions, the likelihood of collisions with residual gas molecules is high, and due to their low mass, the impact of such collisions is elevated.” While the latter point is valid, the first part may require clarification. Since mean free path is inversely proportional to particle size, a low m/z (presumably smaller) ion would not necessarily be more likely to undergo collisions, although the effects of such collisions may indeed be more significant. Please clarify or revise this statement.

We thank the reviewer for highlighting this important nuance between low- and high-m/z ions and the likelihood of collisions. While the mean free path is higher for smaller particles, the distance that low m/z particles travel in the Orbitrap is longer due to their higher frequency. Because of the longer travel path, the number of collisions increases. In response to the reviewer we modified the sentence on page 5, lines 85-86.

-In Figure 4B, the improvement in ion half-life appears to diminish beyond a certain oligomer size. Could the authors comment on the mechanistic basis for this trend? In particular, it would be helpful to provide a more generalizable discussion relating ion size and mean free path at this pressure regime, and how these factors translate into improvements and/or scale with molecular weight.
-Page 14, line 222, similarly notes that the improvement in ion lifetime becomes less pronounced as molecular weight increases. This trend warrants further explanation. Are there specific classes of analytes for which this approach meaningfully extends accessibility, particularly in cases where collisions with background gas would otherwise be prohibitively detrimental?

The shorter ion half-life for the larger oligomers is most likely due to incomplete desolvation, given that we used the same pressure setting and HCD parameters for all IgG oligomers. Measuring all oligomers in parallel requires a trade-off in MS settings (pressure, HCD energy, tuning parameters, etc.), which would only apply in this particular case of measuring a broad range of molecular weights. We added an extra sentence to comment on this on page 14 line 227.

We have also modified a part of the discussion to comment on ion size in relation to collisions with the background gas (page 20 lines 309-313). In previous research, our group has gone more into depth on ion collisions with background gas and added a reference (ref 40).

-In Figure 5, the caption appears to be missing a word in the phrase "residues in red divergence from the suspected binding sequence." Please revise for clarity. In addition, what do the annotations in the figure "(1:73)" and "(1:94)" refer to? Do these represent molar ratios? Please clarify in the figure caption or main text.

We thank the reviewer for catching this typo. We have modified the sentence. Indeed, the ratios represent the molar ratios, we modified the caption accordingly.

-Further to this, according to the methods section, the peptide concentration used (70 μM) is quite high and could potentially promote nonspecific binding or artifacts. The authors should clarify how nonspecific interactions were ruled out. This could be addressed either by providing a quantitative justification (e.g., probability calculations) or by including a peptide titration experiment to demonstrate that the additional binding observed is not nonspecific.

The data in Figure 5 are at high access of the ligand SpA. As also suggested by reviewer 2, we have now added in the revised manuscript a titration experiment which we expect to clarify that we do not see too much nonspecific binding, but do see a real difference in binding of SpA to the two different IgG I and IgG II constructs.

Overall, the manuscript presents a clever hardware modification supported by compelling examples. However, its impact for a broader readership would be strengthened by a clearer articulation of the generalizable advantages of the approach (e.g., the molecular weight range over which it is most beneficial and why that is so).
